# 6 versus 12 months of adjuvant trastuzumab for HER2-positive early breast cancer (PERSEPHONE): 4-year disease-free survival results of a randomised phase 3 non-inferiority trial

**DOI:** 10.1016/S0140-6736(19)30650-6

**Published:** 2019-06-29

**Authors:** Helena M Earl, Louise Hiller, Anne-Laure Vallier, Shrushma Loi, Karen McAdam, Luke Hughes-Davies, Adrian N Harnett, Mei-Lin Ah-See, Richard Simcock, Daniel Rea, Sanjay Raj, Pamela Woodings, Mark Harries, Donna Howe, Kerry Raynes, Helen B Higgins, Maggie Wilcox, Chris Plummer, Janine Mansi, Ioannis Gounaris, Betania Mahler–Araujo, Elena Provenzano, Anita Chhabra, Jean E Abraham, Carlos Caldas, Peter S Hall, Christopher McCabe, Claire Hulme, David Miles, Andrew M Wardley, David A Cameron, Janet A Dunn, PERSEPHONE Steering Committee and Trial Investigators, Roshan Agarwal, Hafiz Algurafi, Rozenn Allerton, Caroline Archer, Anne Armstrong, Catherine Bale, Lisa Barraclough, Urmila Barthakur, Carolyn Bedi, Kim Benstead, David Bloomfield, Rebecca Bowen, Chris Bradley, Jane Brown, Mohammad Butt, Mark Churn, Susan Cleator, Joanne Cliff, Perric Crellin, Margaret Daly, Shiroma De Silva-Minor, Amandeep Dhadda, Omar Din, Sue Down, Helena Earl, David Eaton, Andrew Eichholz, Daniel Epurescu, Chee Goh, Andrew Goodman, Robert Grieve, Maher Hadaki, Catherine Harper-Wynne, Mark Harries, Larry Hayward, Alison Humphreys, Helen Innes, Mariam Jafri, Apurna Jegannathen, Muireann Kelleher, Hartmut Kristeleit, Daniela Lee, Susan Lupton, Carol MacGregor, Zafar Malik, Janine Mansi, Jennifer Marshall, Karen McAdam, Trevor McGolick, Rakesh Mehra, David Miles, Natasha Mithal, Charlotte Moss, Aian Moss, Mukesh Mukesh, Anthony Neal, Daniel Nelmes, Helen Neville-Webbe, Jacqueline Newby, Susan O'Reilly, Peter Ostler, Mojca Persic, Laura Pettit, Sanjay Raj, Fharat Raja, Daniel Rea, Catherine Reed, Anne Rigg, Helen Roe, Nihal Shah, Peter Simmonds, Eliot Sims, Sarah Smith, Nicola Storey, Wendy Taylor, Narottam Thanvi, Karen Tipples, Jayant Vaidya, Mohini Varughese, Anup Vinayan, Nawaz Walji, Simon Waters, Pamela Woodings, Kathryn Wright, Sundus Yahya

**Affiliations:** a Department of Oncology, University of Cambridge, Addenbrooke's Hospital, Cambridge, UK; b Metabolic Research Laboratories, University of Cambridge, Addenbrooke's Hospital, Cambridge, UK; c Cambridge Breast Cancer Research Unit, Cambridge University Hospitals National Health Service (NHS) Foundation Trust, Cambridge, UK; d Cambridge Clinical Trials Unit-Cancer Theme, Cambridge University Hospitals National Health Service (NHS) Foundation Trust, Cambridge, UK; e Department of Oncology, Cambridge University Hospitals National Health Service (NHS) Foundation Trust, Cambridge, UK; f Department of Histopathology, Cambridge University Hospitals National Health Service (NHS) Foundation Trust, Cambridge, UK; g Pharmacy, Cambridge University Hospitals National Health Service (NHS) Foundation Trust, Cambridge, UK; h National Institute for Health Research Cambridge Biomedical Research Centre, Cambridge, UK; i Warwick Clinical Trials Unit, University of Warwick, Coventry, UK; j Department of Oncology, North West Anglia NHS Foundation Trust, Peterborough City Hospital, Peterborough, UK; k Department of Oncology, James Paget University Hospital, Norfolk, UK; l Department of Oncology, Norfolk & Norwich University Hospital, Norwich, UK; m Medical Oncology, Mount Vernon Cancer Centre, Northwood, UK; n Sussex Cancer Centre, Brighton and Sussex University Hospitals NHS, Brighton, UK; o Cancer Research UK Clinical Trials Unit and Institute of Cancer and Genomic Sciences, University of Birmingham, Birmingham, UK; p Department of Oncology, Southampton University Hospital NHS Foundation Trust, Southampton, UK; q Department of Oncology, Royal Derby Hospital, Derby, UK; r Department of Medical Oncology, Guy's and St Thomas' NHS Foundation Trust, London, UK; s Independent Cancer Patients Voice, London, UK; t Department of Cardiology, Newcastle upon Tyne Hospitals NHS Foundation Trust, Newcastle upon Tyne, UK; u Freeman Hospital, Newcastle upon Tyne, UK; v Oncology Global Drug Development, Novartis, Basel, Switzerland; w Cancer Research UK Cambridge Institute, University of Cambridge Li Ka Shing Centre, Cambridge, UK; x Cancer Edinburgh Research Centre, The Institute of Genetics and Molecular Medicine, University of Edinburgh, Western General Hospital, Edinburgh, UK; y Institute of Health Economics, Edmonton, Canada; z Academic Unit of Health Economics, University of Leeds, Leeds, UK; aa Health Economics Group, Institute of Health Research, University of Exeter Medical School, Exeter, UK; ab Research & Development, The NIHR Manchester Clinical Research Facility at The Christie NHS Foundation Trust, Manchester, UK; ac Division of Cancer Sciences, Faculty of Biology, Medicine and Health, ManchesterAcademic Health Science Centre, University of Manchester, Manchester, UK

## Abstract

**Background:**

Adjuvant trastuzumab significantly improves outcomes for patients with HER2-positive early breast cancer. The standard treatment duration is 12 months but shorter treatment could provide similar efficacy while reducing toxicities and cost. We aimed to investigate whether 6-month adjuvant trastuzumab treatment is non-inferior to the standard 12-month treatment regarding disease-free survival.

**Methods:**

This study is an open-label, randomised phase 3 non-inferiority trial. Patients were recruited from 152 centres in the UK. We randomly assigned patients with HER2-positive early breast cancer, aged 18 years or older, and with a clear indication for chemotherapy, by a computerised minimisation process (1:1), to receive either 6-month or 12-month trastuzumab delivered every 3 weeks intravenously (loading dose of 8 mg/kg followed by maintenance doses of 6 mg/kg) or subcutaneously (600 mg), given in combination with chemotherapy (concurrently or sequentially). The primary endpoint was disease-free survival, analysed by intention to treat, with a non-inferiority margin of 3% for 4-year disease-free survival. Safety was analysed in all patients who received trastuzumab. This trial is registered with EudraCT (number 2006–007018–39), ISRCTN (number 52968807), and ClinicalTrials.gov (number NCT00712140).

**Findings:**

Between Oct 4, 2007, and July 31, 2015, 2045 patients were assigned to 12-month trastuzumab treatment and 2044 to 6-month treatment (one patient was excluded because they were double randomised). Median follow-up was 5·4 years (IQR 3·6–6·7) for both treatment groups, during which a disease-free survival event occurred in 265 (13%) of 2043 patients in the 6-month group and 247 (12%) of 2045 patients in the 12-month group. 4-year disease-free survival was 89·4% (95% CI 87·9–90·7) in the 6-month group and 89·8% (88·3–91·1) in the 12-month group (hazard ratio 1·07 [90% CI 0·93–1·24], non-inferiority p=0·011), showing non-inferiority of the 6-month treatment. 6-month trastuzumab treatment resulted in fewer patients reporting severe adverse events (373 [19%] of 1939 patients *vs* 459 [24%] of 1894 patients, p=0·0002) or stopping early because of cardiotoxicity (61 [3%] of 1939 patients *vs* 146 [8%] of 1894 patients, p<0·0001).

**Interpretation:**

We have shown that 6-month trastuzumab treatment is non-inferior to 12-month treatment in patients with HER2-positive early breast cancer, with less cardiotoxicity and fewer severe adverse events. These results support consideration of reduced duration trastuzumab for women at similar risk of recurrence as to those included in the trial.

**Funding:**

UK National Institute for Health Research, Health Technology Assessment Programme.

## Introduction

Trastuzumab delivered with chemotherapy for patients with HER2-positive breast cancer in the metastatic^1^ and adjuvant settings^2^, ^3^, ^4^ resulted in improved treatment outcomes, and long-term follow-up has confirmed these benefits.^5^, ^6^ A 12-month treatment duration with adjuvant trastuzumab was chosen arbitrarily for the pivotal licensing trials^2^, ^3^, ^4^ and, subsequently, became standard. However, results from the FinHer trial,^7^ which randomly assigned patients to adjuvant chemotherapy with or without 9 weeks of concurrent trastuzumab, showed a statistically significant improvement in disease-free survival for patients assigned to trastuzumab (hazard ratio [HR] 0·29 [95% CI 0·13–0·64; p=0·002) and generated considerable interest in the possibility of shorter trastuzumab durations than the standard treatment time. Trastuzumab has well recognised toxic effects, particularly cardiac,^8^, ^9^, ^10^, ^11^ and substantial costs. Studies have been done to assess whether similar outcomes can be achieved with reduced treatment duration. PHARE (France),^12^ PERSEPHONE (UK),^13^ and the HORG study (Greece)^14^ compared 6-month with 12-month trastuzumab treatments. Short-HER (Italy)^15^ and SOLD (international)^16^ compared treatment for 12 months with treatment for 9 weeks (concurrently with docetaxel-first sequenced chemotherapy), and E2198^17^ compared treatment for 12 months with 12-week treatments (concurrently with weekly paclitaxel). Five of these six de-escalation trials were supported wholly or in part by government funding and aimed to discover the optimal balance between efficacy, toxicity, and cost for patients and health services. The PERSEPHONE trial, reported in this Article, aimed to investigate the hypothesis that 6-month adjuvant trastuzumab treatment is non-inferior to the standard 12-month treatment in terms of outcomes, but with reduced toxicity. The trial uses a non-inferiority design^18^ and we report 4-year disease-free survival results, the definitive primary endpoint.

**Research in context**

**Evidence before this study**

We searched PubMed in October, 2006, using search terms “trastuzumab”, “adjuvant”, and “HER2 positive breast cancer”, for papers published in English. We found three randomised controlled trials of adjuvant trastuzumab added to chemotherapy versus chemotherapy alone for patients with HER2-positive early breast cancer. In 2005, two reports established 12-month trastuzumab as the standard of care. The HERA trial examined both 24-month and 12-month trastuzumab compared with a no trastuzumab control group and reported a benefit of disease-free survival at 2 years of 8·4% (95% CI 2·1–14·8) for the 12-month treatment group compared with the no trastuzumab group. The joint analysis of the US studies (NSABP-B31 and NCCTG-N9831) showed a similar hazard ratio (HR) for 12-month trastuzumab and a control group that did not receive this drug, with a benefit of disease-free survival of 11·8% at 3 years. However, in 2006 a smaller study (FinHer) reported just 9-week trastuzumab with chemotherapy versus chemotherapy control, and showed very similar results with a benefit in disease-free survival of 11·7% at 3 years for 9-week trastuzumab. This result prompted a substantial interest in whether duration of trastuzumab could be reduced and a number of trials were started all with a non-inferiority design, to establish whether shorter duration trastuzumab could have similar efficacy but reduced toxicity.

**Added value of this study**

The Persephone trial was a pragmatic study of 6-month versus 12-month trastuzumab, which mapped onto standard practice in the UK and included all patients who were planned to receive adjuvant or neoadjuvant trastuzumab and chemotherapy. Broad eligibility criteria, and in particular the inclusion of all chemotherapy regimens, ensured that results would be directly applicable to similar patients in the clinic. The trial has a non-inferiority design and, with recruitment of over 4000 patients, was powered to test whether 6-month treatment was no worse than 3% less of the standard disease-free survival with 12-month treatment. PERSEPHONE is the largest of the reduced duration, adjuvant trastuzumab trials, it recruited the required number of patients, and accrued the number of events set out in the statistical analysis plan. This trial is the only one to show clear non-inferiority for reduced duration trastuzumab in HER2-positive early breast cancer.

**Implications of all the available evidence**

Available evidence for trastuzumab duration includes the SOLD and Short-HER trials, which compared 9-week trastuzumab with 12-month trastuzumab, and neither trial showed non-inferiority. The HORG trial (n=481) and the PHARE trial (n=3380) compared 6-month with 12-month trastuzumab and also did not show non-inferiority. The PHARE trial was presented in December, 2018, with a median follow-up of 90 months and showed a HR of 1·08 (95% CI 0·93–1·25), which is remarkably similar to the data we report from PERSEPHONE's findings (1·07 [0·93–1·24]). However, the PHARE trial set a 2% non-inferiority margin and HR boundary of 1·15, and, therefore, accordingly cannot claim non-inferiority. During the trial, standard treatments gradually changed in the clinic and PERSEPHONE has captured these data. PERSEPHONE is the only trial to show clear non-inferiority for 6 months of trastuzumab in the early disease setting in the population treated and we believe this result, together with the recent, mature data from PHARE, should signal reduced duration trastuzumab becoming a standard of care in patients with HER2-positive early breast cancer.

## Methods

### Study design

PERSEPHONE was a prospective, multicentre, phase 3 randomised trial to test the hypothesis that 6 months of trastuzumab treatment is non-inferior to the standard 12-month treatment. Patients were recruited in 152 hospitals in the UK. The trial was approved by the Multi-Centre Research Ethics Committee (MREC) on Aug 9, 2007, (07/MRE08/35), and then by Research and Development departments at each participating institution. It was sponsored by Cambridge University Hospital National Health Service Trust and University of Cambridge; and co-ordinated and analysed by the Warwick Clinical Trials Unit at the University of Warwick. The trial was done in accordance with the Declaration of Helsinki and supported by the National Cancer Research Network (number 4078), which funded research personnel in local networks to support the trial. The trial was run under the auspices of an independent data safety and monitoring committee (IDSMC), an independent trial steering committee (TSC), and a trial management group (TMG). Ethical approval is from the MREC. The protocol can be found online on the Warwick Clinical Trial Unit website.

### Participants

Eligible patients, aged 18 years or older, had a histological diagnosis of invasive early breast cancer with overexpression of HER2 receptor, defined according to the American Society of Clinical Oncology and College of American Pathologists guidelines.^19^ All patients had a clear indication for chemotherapy and provided written informed consent. At the beginning of the trial, all patients were randomised before starting trastuzumab. However, in 2009, recruitment numbers remained substantially lower than expected and after discussion between the TMG, TSC, IDSMC, and funders, a protocol amendment (protocol version 3.1; July, 2009) allowed randomisation to occur at any time up to and including the ninth cycle of trastuzumab. All participants were considered medically fit to receive treatment by the responsible clinician. Female patients who were of child-bearing potential were non-pregnant, non-lactating, and agreed to use adequate contraception during treatment. For full eligibility criteria see online protocol.

### Randomisation and masking

**Figure 1:**
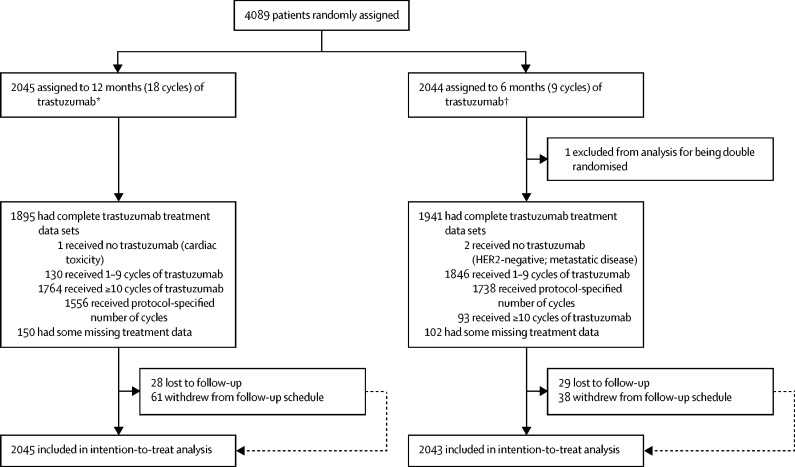
Trial profile *Seven patients were found to be ineligible after randomisation (four had previous cancer or ductal carcinoma in situ treated with surgery and radiotherapy, two were HER2 negative, and one had primary cancer confined to the axilla). †11 patients were found to be ineligible after randomisation (seven had previous cancer or ductal carcinoma in situ treated with surgery and radiotherapy, one was HER2 negative, two had metastatic disease, and one had received >9 cycles of trastuzumab).

The trial was open label and patients were randomly assigned (1:1) to either 12 months (standard 18 cycles) of trastuzumab or 6 months (experimental nine cycles) of trastuzumab (figure 1). Randomisation was done by telephone to the Warwick Clinical Trials Unit, where a central computerised minimisation procedure used the following stratification variables: oestrogen receptor status (positive or negative); chemotherapy type (anthracycline without taxane, anthracycline with taxane, taxane without anthracycline, or neither anthracycline nor taxane); chemotherapy timing (adjuvant or neoadjuvant chemotherapy); and trastuzumab timing (concurrent or sequential).

### Procedures

Trastuzumab was administered every 3 weeks either intravenously or, following a third protocol amendment (version 4.0, published Oct 31, 2013), subcutaneously. Switching from intravenous to subcutaneous route was allowed at clinician discretion. The intravenous loading dose was 8 mg/kg followed by maintenance doses of 6 mg/kg, and the subcutaneous dose was fixed at 600 mg.

After randomisation, patients were followed up routinely at the centre where the patient was recruited or an associated institution with ethical approval for the study. Follow-up was every 12 weeks for the first year after starting trastuzumab and all toxic effects were recorded. Common Toxicity Criteria Adverse Events (CTCAE, version 3) grades were recorded for each trastuzumab cycle after randomisation. Originally, left ventricular ejection fraction (LVEF) assessments every 3 months for up to 12 months after starting trastuzumab were required for all patients. However, in June, 2013, the IDSMC recommended reducing LVEF monitoring to every 4 months, in line with new national guidelines.^20^ Trastuzumab was discontinued if LVEF decreased to lower than 50%; LVEF was reassessed after 6 weeks and 12 weeks. Trastuzumab was restarted if the LVEF recovered; however, if treatment could not be restarted for 12 weeks because of persistently low LVEF, it was stopped permanently. Patients were followed up every 6 months for the second year and annually thereafter, in accordance with standard local practice, and continued for 10 years.

The quality of life assessment schedule, defined by our group for the purposes of the trial, specifies assessments before starting trastuzumab, and then 3, 6, 9, 12, 18, and 24 months later. All patients followed this schedule from the time they entered the trial, completing assessments at the same timepoints in their treatment. Patients who were randomised during their trastuzumab treatment did not have a baseline quality of life assessment and some also did not have the 3-month assessment if randomised after this timepoint. Questions regarding general health and the Euroqol 5D questionnaire 3 level (EQ-5D-3L) were recorded. The health economic analysis will be reported separately.

### Outcomes

The primary endpoint was disease-free survival, which was calculated from the date of diagnostic biopsy to the date of first invasive breast cancer relapse (local or distant) or death, or to date of censor in patients alive and relapse-free. The secondary endpoint of overall survival was also calculated from the date of diagnostic biopsy. Other secondary endpoints were expected incremental cost-effectiveness (health economic analysis), cardiac function as assessed by LVEF during treatment, and analysis of predictive factors for development of cardiac damage. Since randomisation could occur at any time up to and including the ninth cycle of trastuzumab, a prespecified landmark analysis was done from 6 months after the start of trastuzumab. The number of trastuzumab cycles received per patient was recorded, with route of administration and reasons for any deviation from protocol. LVEF measurements were defined as low if results were less than 50% or reported as low without quantification of LVEF. Incidence of clinical cardiac dysfunction, defined as symptoms or signs of congestive heart failure or new cardiac medication, was recorded every 3 months for 12 months. A cardiologist (CP) was a member of the trials group and reviewed the cardiac toxicity together with the Chief Investigator (HME) and other members of the TMG.

Disease-free interval, distant disease-free interval, distant disease-free survival, invasive disease-free survival (including contralateral breast and second primary cancers, according to the STandardized definitions for Efficacy End Points system),^21^ and breast-cancer specific survival were also analysed post hoc.

### Statistical analysis

The trial was designed to assess non-inferiority of the experimental group (6 months of trastuzumab), and the clinically acceptable non-inferiority margin for the 6-month group was defined as being not worse than an absolute value of 3% below the 4-year disease-free survival of the standard group (12 months of trastuzumab). This 3% non-inferiority margin was decided before the start of the trial following consensus from the trial development group together with the patient and public involvement group. Data from adjuvant trastuzumab trials at the time^2^, ^3^, ^4^ estimated the 4-year disease-free survival for patients treated with 12 months of trastuzumab to be 80%. Consequently, 4000 patients (2000 in each group) were required to show the non-inferiority of 6-month trastuzumab treatment with a 3% non-inferiority margin of the 4-year disease-free survival, and 5% one-sided significance and 85% statistical power. This calculation assumes a 4-year recruitment period, an additional 5-year follow-up period, and 4% of patients being lost to follow-up. Survival curves were plotted using the Kaplan-Meier method and the HR between the two groups was estimated using a Cox's proportional hazards model containing only the trial treatment effect, after assessment of the proportionality of hazards using log-log plots. The upper limit of the HR required to show the 3% non-inferiority was only to be calculated at the time of analysis and based on the disease-free survival in the 12-month group observed at the time of analysis. As described by Mauri and D'Agostino,^22^ if the upper limit of the 90% CI (the 95th percentile) of the estimated HR was less than the relevant 3% absolute non-inferiority limit, then the experimental group (6-month trastuzumab) would be regarded as non-inferior.

Warwick Clinical Trials Unit analysed all the data using SAS (version 9.4) software. The IDSMC-approved statistical analysis plan stated that the event-driven primary endpoint analysis required 500 disease-free survival events to have occurred. At this point, the relevant non-inferiority limits in terms of HR for 3% non-inferiority in disease-free survival were calculated using the observed 4-year disease-free survival events in the standard, 12-month group. The statistical analysis plan included a secondary analysis adjusting for stratification factors within the Cox model and also the presentation of treatment effect on disease-free survival for stratification variables and baseline prognostic factors using HR plots with the test for interaction or heterogeneity using Cochran's Q.^23^ To remove the effect of timing of randomisation, the statistical analysis plan also defined an exploratory landmark analysis for patients alive and disease-free 6 months after starting trastuzumab. All randomly assigned patients were included in all outcome analyses, and all patients who started trastuzumab were included in all safety analyses. All analyses were done on an intention-to-treat basis because the trial aimed to compare the duration of trastuzumab treatments in routine clinical practice. Treatment analyses were done for all patients with available data. PERSEPHONE is registered with EudraCT (number 2006–007018–39), International Standard Randomised Controlled Trial (number ISRCTN 52968807), and ClinicalTrials.gov (number NCT00712140).

### Role of the funding source

The funder of the study had no role in study design, data collection, data analysis, data interpretation, or writing of the report. The corresponding author had full access to all the data and, along with LH and JAD, had final responsibility for the decision to submit for publication with the agreement of all the authors and the IDSMC.

## Results

Between Oct 4, 2007, and July 31, 2015, a total of 4089 patients were randomly assigned by 210 clinicians at 152 sites in the UK (figure 1). One double randomisation reduced the analysis set to 4088 patients. 18 patients were deemed ineligible after randomisation, mainly because of previous cancers or ductal carcinoma in situ treated with radiotherapy and surgery. Patient characteristics were similar between the two groups (table 1). Randomisation before starting trastuzumab occurred in 1786 [44%] of 4088 patients and the timings for those randomly assigned after the first cycle of trastuzumab are shown in the appendix (p 1).

**Table 1:** Baseline characteristics of trial participants

	**12-month group (n=2045)**	**6-month group (n=2043)**
Oestrogen receptor status*		
Negative	633 (31%)	632 (31%)
Positive	1412 (69%)	1411 (69%)
Chemotherapy type*		
Anthracycline-based	854 (42%)	846 (41%)
Taxane-based	200 (10%)	203 (10%)
Anthracycline-based and taxane-based	989 (48%)	991 (49%)
No taxane and no anthracycline	2 (<1%)	3 (<1%)
Chemotherapy timing*		
Adjuvant	1737 (85%)	1731 (85%)
Neoadjuvant	308 (15%)	312 (15%)
Trastuzumab timing*		
Concurrent	951 (47%)	952 (47%)
Sequential	1094 (53%)	1091 (53%)
**Age at randomisation, years**		
Median (range)	56 (23–82)	56 (23–83)
<35	50 (2%)	45 (2%)
35–49	552 (27%)	557 (27%)
50–59	608 (30%)	656 (32%)
≥60	835 (41%)	785 (38%)
**Nodal status at surgery (patients who received adjuvant therapy)**		
Negative	1003/1737 (58%)	1019/1731 (59%)
1–3 nodes positive	479/1737 (28%)	486/1731 (28%)
4+ nodes positive	244/1737 (14%)	211/1731 (12%)
Unknown	11/1737 (<1%)	15/1731 (<1%)
**Tumour size (patients who received adjuvant therapy)**†		
≤2 cm	824/1737 (47%)	807/1731 (47%)
>2 and ≤5 cm	778/1737 (45%)	786/1731 (45%)
>5 cm	87/1737 (5%)	83/1731 (5%)
Unknown	48/1737 (3%)	55/1731 (3%)
**Tumour grade**‡		
I (well differentiated)	29 (1%)	34 (2%)
II (moderately differentiated)	628 (31%)	642 (31%)
III (poorly differentiated)	1322 (65%)	1297 (63%)
Unknown	66 (3%)	70 (3%)
**Ethnicity**		
White	1658 (81%)	1648 (81%)
Asian	57 (3%)	52 (3%)
Black	52 (3%)	45 (2%)
Other	17 (1%)	21 (1%)
Unknown	261 (13%)	277 (14%)
**Menopausal status before chemotherapy**		
Premenopausal	567 (28%)	580 (28%)
Perimenopausal	110 (5%)	150 (7%)
Postmenopausal	1144 (56%)	1070 (52%)
Not assessable or not available	224 (11%)	243 (12%)
**Reported previous use of cardiac medication**		
Yes	44 (2%)	55 (3%)
No	2001 (98%)	1988 (97%)
**HER2 immunohistochemistry score and FISH positivity**		
3+	1460 (71%)	1487 (73%)
2+ and FISH positive	540 (26%)	497 (24%)
Not available	45 (2%)	59 (3%)

Data are n (%), unless otherwise specified. Six men are included in the 4088 patients, four in the 12-month group and two in the 6-month group. FISH=fluorescence in-situ hybridisation.

* Stratification variable.

† Size of the largest invasive tumour at diagnosis.

‡ Grade of the largest invasive tumour at diagnosis.

The majority of patients (69%) had oestrogen receptor-positive tumours. Among patients who received neoadjuvant chemotherapy (n=620), the majority (553 [89%]) received anthracycline and taxane, 53 (9%) anthracycline without taxane, and 14 (2%) taxane without anthracycline; for patients who received adjuvant chemotherapy (n=3468), less than half received anthracycline and taxane (1427 [41%]) or anthracycline without taxane (1647 [47%]), and 389 (11%) received taxane without anthracycline (appendix p 1). UK standard practice gradually changed during trial recruitment with a steady increase in anthracycline and taxane combinations, trastuzumab commencing concurrently with taxanes but not anthracyclines, taxane-based treatment without anthracyclines, and neoadjuvant timing (appendix p 2). Patients given trastuzumab and chemotherapy concurrently compared with sequentially, in the adjuvant group, were more often node positive (749 [53%] of 1412 patients *vs* 671 [33%] of 2056, p<0·0001), had larger tumours (>2 cm; 777 [55%] of 1412 patients *vs* 957 [47%] of 2056 patients, p<0·0001); in the whole group, patients more often received neoadjuvant treatment (491 [26%] of 1903 patients *vs* 129 [6%] of 2185 patients, p<0·0001) and had shorter median duration of follow-up (4·5 years [IQR 3·3–5·9] *vs* 5·8 years [4·5–7·4]; appendix pp 2–4). More than half of the patients receiving adjuvant chemotherapy were node negative (2022 [58%] of 3468 participants) and 1631 [47%] of 3468 patients in this group had tumours smaller than 2 cm (table 1).

Complete trastuzumab administration details are available for 3836 (94%) of 4088 patients: 1895 (93%) of 12-month treatment patients and 1941 (95%) of 6-month treatment patients (figure 1). 40 530 (82%) of 49 632 trastuzumab cycles were administered intravenously and 9102 (18%) of 49 632 cycles subcutaneously. In total, 3294 (86%) of 3836 patients received the protocol-specified number of trastuzumab cycles: 1556 (82%) of 1895 patients assigned to 12-month treatment who had available treatment data and 1738 (90%) of 1941 patients assigned to 6-month treatment with available treatment data. The most common reasons for early treatment cessation were cardiac toxicity (146 [8%] of 1894 patients in the 12-month group, 61 [3%] of 1939 patients in the 6-month group) and patient request (91 [5%] of 1894 patients in the 12-month group, 24 [1%] of 1939 patients in the 6-month group; figure 1). Delays occurred in 3631 (7%) of 49 632 cycles (appendix p 4), the main reasons being holidays, sepsis or infection, and cardiotoxicity. The most common chemotherapy treatments (known for 3959 patients: 1985 patients in the 12-month group and 1974 patients in the 6-month group), were fluorouracil, epirubicin, and cyclophosphamide, with docetaxel (811 [41%] of 1985 patients in the 12-month group *vs* 790 [40%] of 1974 in the 6-month group), and without docetaxel (548 [28%] of 1985 *vs* 567 [29%] of 1974).

At database lock on April 17, 2018, with a median follow-up of 5·4 years (IQR 3·6–6**·7**) for both treatment groups and 3588 (96%) of 3753 alive patients followed up for at least 2 years, 335 deaths had been reported (156 [8%] of 2045 patients in the 12-month group and 179 [9%] of 2043 patients in the 6-month group), the majority (272 [81%] of 335 patients: 129 [83%] of 156 in the 12-month group and 143 [80%] of 179 in the 6-month group) due to breast cancer (table 2). Local or distant relapse occurred in 452 (11%) of 4088 patients, with distant metastases in 373 patients (183 [9%] of 2045 in the 12-month group *vs* 190 [9%] of 2043 patients in the 6-month group). These metastases occurred in the liver (81 [44%] of 183 patients *vs* 79 [42%] of 190 patients), bone (61 [33%] patients *vs* 81 [43%] patients), lung (72 [39%] patients *vs* 67 [35%] patients), and brain (38 [21%] patients *vs* 40 [21%] patients). A relapse or death was reported for 512 (13%) patients overall.

**Table 2:** Details of events

		**12-month group (n=2045)**	**6-month group (n=2043)**
Death		156 (8%)	179 (9%)
	Breast cancer listed as a cause	129 (6%)	143 (7%)
	Breast cancer not listed as a cause, but a local or distant relapse reported	2 (<1%)	6 (<1%)
	Breast cancer not listed as a cause and no local or distant relapse reported	25 (1%)	30 (1%)
Relapse*		218 (11%)	234 (11%)
	Local relapse	79 (4%)	77 (4%)
	Distant relapse	183 (9%)	190 (9%)
Relapse or death		247 (12%)	265 (13%)
Second primary		58 (3%)	52 (3%)

Data are n (%).

* Patients can have both a local and distant relapse recorded.

**Figure 2:**
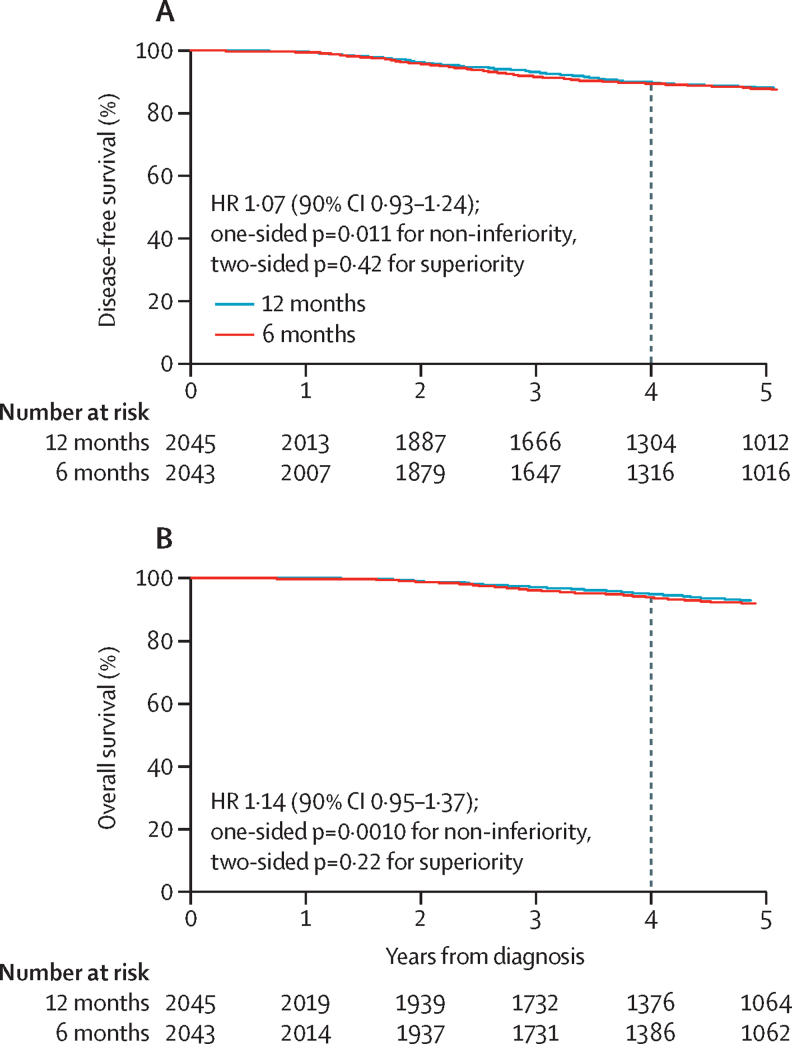
Kaplan-Meier plots of disease-free survival (A) and overall survival (B) for 6-month and 12-month trastuzumab treatment groups

The 4-year disease-free survival in the 12-month group was 89·8% (95% CI 88·3–91·1) and 89·4% (87·9–90·7) in the 6-month group (figure 2A), showing a 0·4% absolute difference in disease-free survival between treatment groups at 4 years. Thus, with the non-inferiority margin of 3%, the non-inferiority limit for the HR was set at 1·32. The HR for relapse or death with 6 months compared with 12 months trastuzumab was 1·07 (90% CI 0·93–1·24, non-inferiority p=0·011); this outcome met the prespecified definition of non-inferiority. The two-sided p value for difference between treatments (superiority) was 0·42. Adjustment for all stratification factors gave the same results with an HR of 1·07 (90% CI 0·93–1·24; non-inferiority p=0·0097). Analysis of overall survival (figure 2B) also met the prespecified definition of non-inferiority (4-year overall survival of 94·8% [95% CI 93·7–95·8] in the 12-month group and 93·8% [92·6–94·9] in the 6-month group), showing a 1% absolute difference in overall survival between treatment groups at 4 years. The non-inferiority limit for the HR was set at 1·60. The HR for overall survival was 1·14 (90% CI 0·95–1·37, non-inferiority p=0·0010). The two-sided p value for difference between treatments was 0·22 and adjustment for all stratification factors resulted in similar results (HR 1·13 [90% CI 0·94–1·35]).

**Figure 3:**
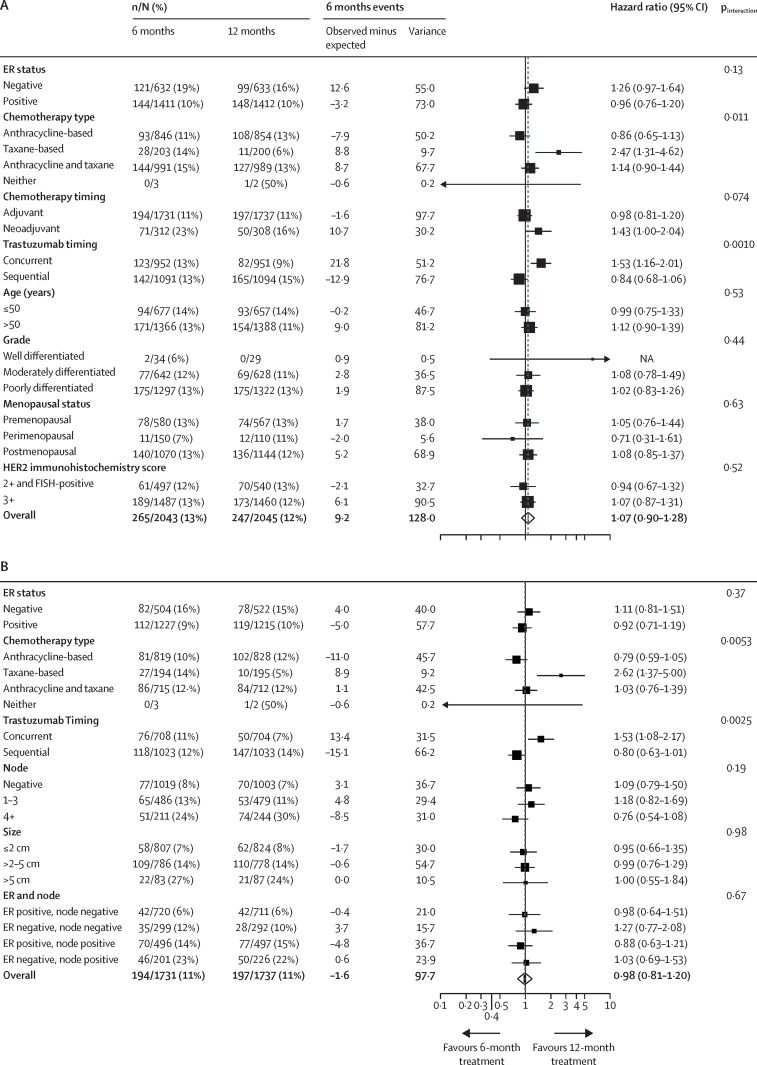
Forest plots of disease-free survival for all patients (A) and patients who received adjuvant treatment (B) FISH=fluorescence in-situ hybridisation. ER=oestrogen receptor.

Forest plots for disease-free survival that included all patients (figure 3A) showed heterogeneity for chemotherapy type (p=0·011), predominantly driven by the small number of events in the taxane-only group, in which most patients received docetaxel with cyclophosphamide.^24^ The timing of trastuzumab relative to chemotherapy (concurrent or sequential) showed heterogeneity between groups (p=0·0010), favouring the 12-month treatment for patients receiving concurrent trastuzumab. Forest plots for overall survival (appendix p 7) also showed heterogeneity for concurrent and sequential treatments; they additionally showed heterogeneity for oestrogen receptor-status (p=0·019), with improved outcomes for 12-month trastuzumab treatments for patients with oestrogen receptor-negative disease. No heterogeneity was observed for age, grade, menopausal status, or HER2 score (immunohistochemistry score 3+, or score 2+ with fluorescence in-situ hybridisation-positive) for disease-free survival or overall survival. Exploratory forest plots for patients who received adjuvant therapy only (figure 3B; appendix p 8) showed similar results, with no heterogeneity for node status, size, and combined oestrogen receptor and node status.

**Figure 4:**
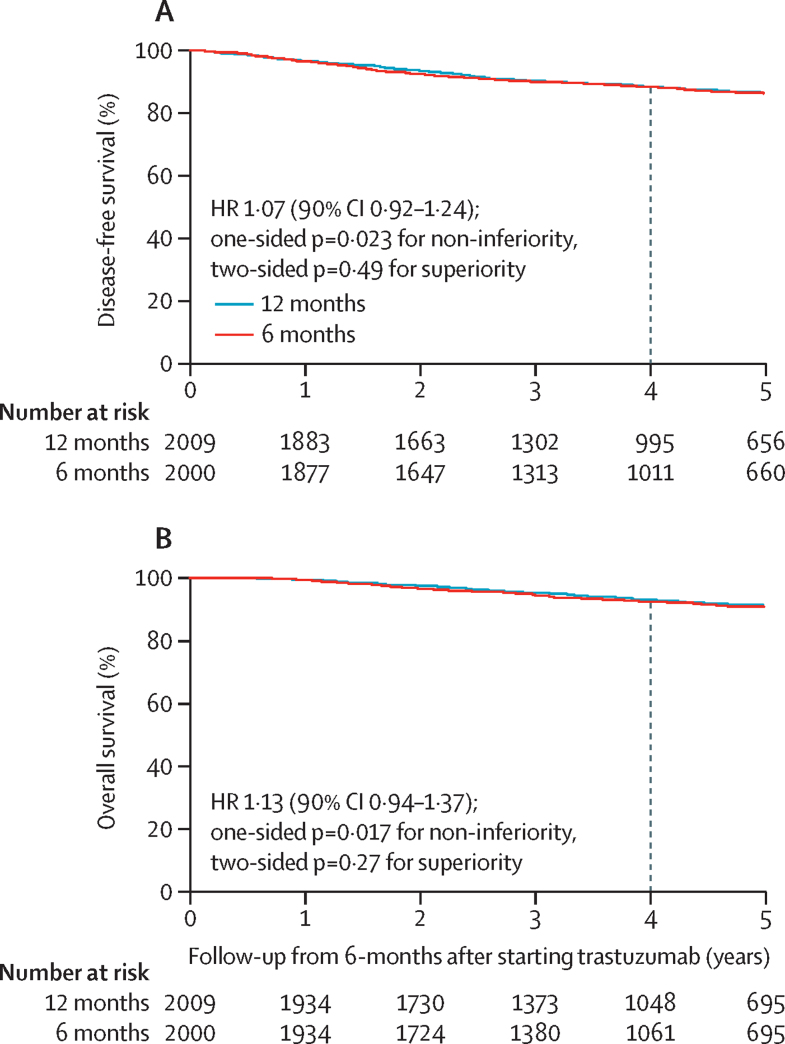
Kaplan-Meier plots of landmark analysis from 6-months after starting trastuzumab treatment (A) Disease-free survival. (B) Overall survival.

The landmark analysis of disease-free survival and overall survival included 4009 patients (2009 patients in the 12-month group and 2000 patients in the 6-month group) who remained alive and disease-free 6 months after starting trastuzumab. Kaplan-Meier (figure 4) and forest plots (appendix pp 9–12) for landmark disease-free survival and overall survival showed similar results to those observed for disease-free survival and overall survival. Landmark 4-year disease-free survival was 88·3% for the 12-month group and 88·2% for the 6-month group (absolute difference 0·1%). Therefore, the non-inferiority limit for the HR was set at 1·28 and, with a calculated HR of 1·07 (90% CI 0·92–1·24), this outcome met the prespecified definition of non-inferiority (non-inferiority p=0·023). Landmark 4-year overall survival was 92·8% for the 12-month group and 92·2% for the 6-month group (absolute difference 0·6%). Thus, the non-inferiority limit for the HR was set at 1·44 and, with a calculated HR of 1·13 (90% CI 0·94–1·37), this outcome met the prespecified definition of non-inferiority (non-inferiority p=0·017). Congruent results were found for disease-free interval, distant disease-free interval, distant disease-free survival, invasive disease-free survival, and breast-cancer specific survival analyses (data not shown).

During the 12-month period after starting trastuzumab, a higher proportion of 12-month patients (459 [24%] of 1894 patients) than 6-month patients (373 [19%] of 1939 patients, p=0·00020) reported at least one adverse event of severe grade (ie, CTCAE ≥3, or CTCAE=2 for palpitations; table 3). The excesses were in cough (82 [4·3%] of 1894 patients in the 12-month group *vs* 44 [2·3%] of 1939 patients in the 6-month group, p=0·00049), palpitations (91 [4·8%] *vs* 52 [2·7%], p=0·00072), fatigue (226 [11·9%] *vs* 167 [8·6%], p=0·00086), pain (98 [5·2%] *vs* 62 [3·2%], p=0·0029), chills (67 [3·5%] *vs* 40 [2·1%], p=0·0075), muscle or joint pain (215 [11·4%] *vs* 174 [9·0%], p=0·017), and nausea (35 [1·9%] *vs* 20 [1·0%], p=0·047; appendix p 13). These were seen predominantly during the 7–12-month period. Similarly, 67 serious adverse reactions were reported in 64 patients in the 12-month group and 34 events in 29 patients in the 6-month group; the excesses seen during the 7–12 month period. Clinical cardiac dysfunction was reported more commonly in the 12-month group (224 [11%] of 1968 patients) than in the 6-month group (155 [8%] of 1994 patients; p=0·00014; table 3). A small absolute difference in clinical cardiac dysfunction rates was observed in the first 6 months (p=0·018), with a larger difference during the 7–12-month period (p=0·00020). Trastuzumab was stopped early because of cardiac toxicity in 146 (8%) of 1894 patients in the 12-month group and 61 (3%) of 1939 patients in the 6-month group (p<0·0001).

**Table 3:** Adverse events and cardiac monitoring over the two 6-month periods

		**12-month group**			**6-month group**		
		Total	Months 1–6	Months 7–12	Total	Months 1–6	Months 7–12
Adverse event with severe* CTCAE grade		459/1894 (24%)	350/1894 (18%)	259/1764 (15%)	373/1939 (19%)	370/1939 (19%)	8/93 (9%)
Serious adverse reaction to trastuzumab†		64/2044 (3%)	39/2044 (2%)	25/2019‡ (1%)	29/2041 (1%)	28/2041 (1%)	2/2015‡ (0·1%)
Clinical cardiac dysfunction§		224/1968 (11%)	164/1968 (8%)	157/1936 (8%)	155/1994 (8%)	126/1994 (6%)	96/1894 (5%)
Stopped trastuzumab permanently due to cardiac toxicity		146/1894 (8%)	63/1894 (3%)	83/1764 (5%)	61/1939 (3%)	60/1939 (3%)	1/93 (1%)
Cardiac death¶		7/2044 (<1%)	0/2044	0/2019‡	4/2041 (<1%)	0/2041	0/2015‡
Cardiac death related to trastuzumab¶		0/2044	0/2044	0/2019‡	0/2041	0/2041	0/2015‡
Low LVEF‖		228/2040 (11%)	148/2040 (7%)	151/1938 (8%)	176/2038 (9%)	146/2038 (7%)	84/1749 (5%)
Substantial falls in LVEF							
	Absolute decrease of ≥10% from baseline to <50%	163/1959 (8%)	98/1950 (5%)	102/1873 (5%)	132/1959 (7%)	102/1954 (5%)	60/1693 (4%)
	LVEF <50% after a baseline of ≥59%	108/1959 (6%)	63/1950 (3%)	71/1873 (4%)	86/1959 (4%)	70/1954 (4%)	32/1693 (2%)

Data are n/N (%). CTCAE=Common Terminology Criteria for Adverse Events. LVEF=left ventricular ejection fraction.

* CTCAE grade of 3 or more, or grade of 2 for palpitations.

† Denominators exclude the three patients known not to have received trastuzumab.

‡ Denominators reduced because of either deaths or withdrawal of consent for follow-up within the first 6 months.

§ Clinical cardiac dysfunction is defined as symptoms of cardiac disease, signs of congestive heart failure, use of new medication for cardiac disease, or a combination of these factors.

¶ 11 deaths were reported to have a cardiac cause, either first cause or contributory; none occurred during the first 12 months after starting trastuzumab treatment; nine patients died without metastatic disease and two had metastatic disease; in all cases, trastuzumab was considered unrelated or unlikely to be related with the cardiac problems.

‖ Low LVEF was defined as ejection fraction of less than 50% or unknown ejection fraction but classified on report as not normal.

In total, 19 414 measurements of LVEF were made in 4078 patients: 10 162 in 2040 12-month patients and 9252 in 2038 6-month patients. During the first 6 months of treatment, proportions of patients with low LVEF were 7% in both groups (p=0·96). However, during months 7–12, this proportion increased for the 12-month group (8%) but decreased for the 6-month group (5%; p=0·0003 for difference between groups). During months 7–12, substantial decreases of LVEF to less than 50% after a baseline of 59% or more occurred in 71 (4%) of 1873 patients in the 12-month and 32 (2%) of 1693 patients in the 6-month group (p=0·0010). 11 deaths were considered cardiac (primary or contributory cause); however, none occurred during trastuzumab treatment and none were considered to be associated with trastuzumab by the TMG (appendix p 5, 6). Comparing the 1786 (44%) of 4088 patients randomised into the trial before the start of trastuzumab with all patients, the comparison of toxicity between the two treatment groups was broadly similar, although toxicity, including cardiotoxicity, showed marginally higher proportions of patients reporting these effects in both 6-month and 12-month treatment groups for patients randomised before the start of trastuzumab (appendix p 6).

3910 patients (1960 patients in the 12-month group, 1950 patients in the 6-month group) participated in the substudy assessing quality of life. In both groups, feelings of general health declined during the first 3 months of trastuzumab (appendix p 14), when 1816 (46%) of 3910 patients were receiving concurrent chemotherapy, and then steadily improved after completion of treatment. The EQ-5D-3L health state remained steady from baseline to 3 months for both randomised groups and appeared to slowly increase after this period, occurring slightly later for 12-month patients (appendix p 15).

## Discussion

PERSEPHONE showed non-inferiority for 6 months of trastuzumab compared with 12 months. Our definition of non-inferiority was no worse than 3% absolute below the standard group's 4-year disease-free survival, and the non-inferiority limit was thus calculated as an HR of less than 1·32. Notably, the upper confidence limit of the HR was 1·24, which is statistically significantly below this non-inferiority boundary (non-inferiority p=0·011). This finding reflects that, although the non-inferiority boundary was set at 3%, the actual point estimate reduction observed was very small at 0·4% for 4-year disease-free survival and 0·1% for the landmark 4-year disease-free survival.

Other outcome analyses, including overall survival, landmark overall survival 6 months after the start of trastuzumab, and sensitivity analyses for other survival endpoints, were all congruent, showing the non-inferiority of 6-months treatment. In addition, fewer cardiac events and other toxic effects occurred with 6-month treatment and, therefore, the balance of risk and benefit favours shorter treatment. Notably, the patient population in PERSEPHONE, mapping onto standard practice in the clinic, had a substantially better profile of standard prognostic factors than patients treated in the original adjuvant trastuzumab trials.^2^, ^3^, ^4^, ^5^ The trial included 69% patients with oestrogen receptor-positive tumours compared with 36–54% in registration studies; 58% patients were node negative compared with 7–33%; and 47% had tumours of 2 cm or less compared with 35–40%. These standard prognostic factors are similar to those for patients entered into PHARE^12^ and the other non-inferiority trials.^15^, ^16^ PERSEPHONE recruited the number of patients specified in the statistical plan and with an event-driven analysis was sufficiently powered to meet the primary endpoint. To our knowledge, PERSEPHONE is the largest trastuzumab duration comparison study for early breast cancer and the only one to show non-inferiority for the primary endpoint of reduced duration adjuvant trastuzumab.

Before the set-up of the trial, the PERSEPHONE TMG and the patient and public involvement group agreed that an absolute difference up to 3% for the 6-month treatment was considered acceptable by clinicians and patients. This margin is commonly used in non-inferiority trials in oncology, including in the TAILORx study,^26^ published in 2018. Funders (which provided national and international peer review for the trial), National Research Ethics Committee, and each recruiting centre endorsed the view that showing this non-inferiority margin would be important and, if proven, would be potentially practice changing. In the PERSEPHONE statistical analysis plan, we planned to calculate the HR limit of non-inferiority using the observed 4-year disease-free survival in the standard group at the time of the primary endpoint analysis. This statistical analysis plan was approved by the IDSMC as most appropriate for the study.

Two other randomised studies compared treatment for 6 months with 12 months. The HORG^14^ study included only 481 patients and used a non-inferiority margin of 8% using dose-dense fluorouracil, epirubicin, and cyclophosphamide followed by docetaxel as chemotherapy with 6 months or 12 months of trastuzumab commencing concurrently with docetaxel. This relatively small trial with low power did not show non-inferiority for 3-year disease-free survival. The PHARE trial^12^ had an original recruitment target of 7000 patients with a 2% non-inferiority margin. The trial included fewer patients than originally planned (n=3384) and reported an early primary endpoint of disease-free survival at 2 years on the advice of the IDSMC. The HR non-inferiority limit was prespecified at 1·15 on the expected standard group for a disease-free survival of 85% at 2 years. The trial reported an HR of 1·28 (95% CI 1·05–1·56; non-inferiority p=0·29) and, therefore, did not show non-inferiority. The longer-term results from PHARE^27^ were presented in December, 2018, after a median of 7·5 years and the HR was 1·08 (95% CI 0·93–1·25; non-inferiority p=0·39) with the upper confidence limit still exceeding the HR limit of 1·15. Therefore, the conclusion remained the same: non-inferiority was not confirmed. Although the HR at 7·5 years median follow-up is remarkably similar to that measured in the PERSEPHONE trial, each trial is correctly reported according to its own prespecified statistical analysis plan, and hence reported conclusions are different. The PERSEPHONE and PHARE trials groups established a collaboration at the start of the trials for an individual patient data meta-analysis or joint analysis, which will provide larger numbers for exploratory subgroup analyses.

Long-term follow-up of patients within the PERSEPHONE trial is planned. The importance of long-term follow-ups in trials of HER2-positive breast cancer has been emphasised by several studies in which results have changed with time. The longer-term follow-up of PHARE^27^ showed a reduction in the HR for disease recurrence or death compared with the 2-year results. In contrast, the FinHer study,^28^ which provided the stimulus for all the reduced duration trials, showed with longer follow-up^28^ less effect in terms of the HR for disease recurrence or death, which increased from 0·42 at 3 years to 0·65 with a median follow-up of more than 5 years. In addition, a presentation^29^ in 2018 from a combined analysis of the N9831 and NSABP B31 studies showed that the risk of recurrence after 5 years was higher in patients with oestrogen receptor-positive and HER2-positive breast cancer than those with oestrogen receptor-negative and HER2-positive disease; this finding is relevant for PERSEPHONE because of the inclusion of a high proportion of oestrogen receptor-positive patients. Longer follow-up in the PERSEPHONE trial will also be particularly important because changes in standard treatments during the trial mean that for concurrent, anthracycline with taxane-based, and neoadjuvant treatments, the follow-up will be on average shorter than for those receiving sequential, anthracycline-based and adjuvant treatment.

Two trials tested 9 weeks concurrent treatment versus 12 months and neither showed non-inferiority. SHORTHer^15^ randomly assigned 1253 patients and the non-inferiority limit for a less than 3% margin below standard treatment was an HR of less than 1·29 for the primary endpoint of disease-free survival at 5 years; the trial HR was 1·15 (90% CI 0·91–1·46). SOLD^16^ randomly assigned 2176 patients and the non-inferiority limit for a margin of less than 4% was an HR of less than 1·385 for the primary endpoint of disease-free survival at 5 years; the trial HR was 1·39 (90% CI 1·12–1·72). One potential explanation is that the total dose of trastuzumab in the 9-week group was 20 mg/kg, which is substantially less than the 56 mg/kg in PERSEPHONE, PHARE,^12^ and HORG.^14^ This total dose might be insufficient to produce a non-inferior outcome compared with 12 months, even when used concurrently with docetaxel immediately after surgery.

Heterogeneities between prespecified stratification subgroups are seen in the PERSEPHONE trial. For disease-free survival, there is apparent heterogeneity (p=0·0010) for timing of trastuzumab and chemotherapy: patients receiving concurrent treatment appeared to benefit more from standard 12 months of trastuzumab. This result is intriguing because we anticipated that concurrent rather than sequential timing of trastuzumab and chemotherapy would show non-inferiority on the basis of evidence of synergy of concurrent treatment from in-vitro data,^30^ and metastatic^1^ and adjuvant^7^, ^12^, ^31^ clinical trials. We cannot readily explain the heterogeneity measured in our trial for trastuzumab scheduling and duration, but it is important to note that the decision to use concurrent or sequential treatment was selected by investigators and not randomised. Given that patients treated with concurrent chemotherapy also generally had more high-risk features, it is not clear whether the observed heterogeneity is due to the treatment schedule itself, the type of chemotherapy used, or whether it reflects the underlying risk of relapse. However, although the trial was stratified by concurrent and sequential chemotherapy and subgroups are balanced in terms of numbers of patients receiving either 6 or 12 months trastuzumab, these subgroups are smaller and, therefore, lack statistical power. All patients in the concurrent group received either anthracycline and taxane combinations or taxane-based chemotherapy without anthracyclines. HERA^2^, ^32^ is the only trial reporting data for an exploratory subgroup analysis of a non-randomised comparison of sequential administration of trastuzumab after anthracycline and taxane, with trastuzumab after anthracycline without taxane chemotherapy Although trastuzumab appeared to be less efficacious in the anthracycline and taxane group than in the anthracycline alone group, this interaction was not found to be statistically significant. Although we acknowledge that any possible interaction between concurrent and sequential trastuzumab with chemotherapy might raise some concern, this subgroup analysis lacks statistical power for non-inferiority. The heterogeneity showed for different chemotherapy backbones is driven mainly by the taxane without anthracycline group and this result should be interpreted with caution given the small size of this group and the very small number of events. For overall survival in the PERSEPHONE trial, heterogeneity was also shown for oestrogen receptor status; patients with oestrogen receptor-negative disease appeared to benefit more from 12 months trastuzumab, which is perhaps not surprising given the increased risk of relapse in this group.

PERSEPHONE mapped onto standard practice in the UK and study strengths are broad inclusion criteria, which allowed recruitment of a large number of patients in routine clinics and ongoing recruitment as standard chemotherapy regimens and trastuzumab timing changed. The limitations of this design include the potential for complex interactions between trastuzumab duration and prognostic factors, the changing standard practice over the 8 years of the trial, the variable timing of randomisation potentially introducing ascertainment bias, and selection of chemotherapy and trastuzumab timing according to perceived risk by the investigators (ie, concurrent preferred in high-risk patients; appendix pp 2–4). An additional limitation that must be considered is that the analyses were done according to the intention-to-treat principle. Although we believe that this analysis is the most appropriate to use, ensuring unbiased estimates, it can potentially underestimate differences between treatment groups and thus drive the results towards non-inferiority. This limitation is a concern particularly when adherence to treatment is low, differential loss to follow-up occurs, or both. However, in the PERSEPHONE trial, adherence to protocol-mandated treatment is high and loss to follow-up is low.

All reduced-duration trastuzumab trials have shown less cardiotoxicity for the shorter duration.^11^, ^15^, ^16^ In addition, the HERA trial showed that 24 months of trastuzumab further increased rates of cardiotoxicity without improving cancer outcomes.^5^, ^9^ As part of the translational programme in PERSEPHONE, over 80% of patients have donated blood samples that will be part of a genome-wide association study of cardiotoxicity with investigation of interaction with duration of treatment.

Substantial changes to the management of HER2-positive early breast cancer over the past 13 years have occurred, since the concept for the PERSEPHONE trial was developed alongside the other duration trials. The trial was designed to be pragmatic, map onto standard practice, which allowed patients to continue to be enrolled since standard chemotherapy regimens and trastuzumab timings changed. Although this approach is an advantage for trial recruitment and ensures contemporaneity, one of the limitations of this design is that there will not be sufficient power in different subgroups to confirm non-inferiority. Following the report of N9831 in 2011,^31^ which showed superiority of concurrent trastuzumab and taxane chemotherapy over sequential trastuzumab, the use of concurrent treatments increased. Until 2011, the majority of patients in the UK received standard anthracycline-based regimens with sequential trastuzumab similar to those used successfully in HERA.^2^ The docetaxel, carboplatin, and herceptin regimen from the BCIRG 006 study^4^ and the adjuvant paclitaxel and trastuzumab regimen^33^ which avoid anthracyclines, are now widely used in North America and are gaining acceptance in Europe, including the UK. The adjuvant paclitaxel and trastuzumab regimen was developed in particular for patients with low-risk node-negative breast cancer and tested in a phase 2 non-randomised study.^33^ Only 403 (10%) patients in our study, who were enrolled mostly towards the end of the recruitment period, received non-anthracycline containing regimens, in whom very few events have been reported; therefore, no conclusions can be drawn about the effect of a short-duration trastuzumab treatment in combination with a non-anthracycline regimen.

Additional advances in the management of HER2-positive early breast cancer warrant consideration. Trials of neoadjuvant therapy can provide personalised HER2-directed therapies with potential for both escalation and de-escalation strategies. Use of dual anti-HER2 therapy (trastuzumab and pertuzumab) with chemotherapy has been increasing in the neoadjuvant setting following the Neo-SPHERE trial,^34^, ^35^ which showed improved pathological complete response and disease-free survival. In the Katherine trial,^36^ published in 2019, trastuzumab emtansine was tested against trastuzumab after the combination of neoadjuvant chemotherapy and anti-HER2 therapy did not result in a pathological complete response. The estimated percentage of patients who were free of invasive disease at 3 years was 88·3% in the trastuzumab emtansine group and 77·0% in the trastuzumab group. Invasive disease-free survival was significantly higher in the T-DM1 group than in the trastuzumab group (HR for invasive disease or death 0·50 [95% CI 0·39–0·64], p<0·001).^36^ This significant improvement in outcomes is likely to lead to an appropriate escalation of standard treatment in the post-neoadjuvant setting, at least for patients who do not achieve pathological complete response with neoadjuvant treatment. However, patients who do achieve a pathological complete response could be considered for trials that are planned for de-escalation of HER2 therapy. The APHINITY trial^37^ reported improved outcomes for the addition of 12-month adjuvant pertuzumab to 12-month trastuzumab treatment. The reduction in disease recurrence was relatively small, albeit statistically significant (HR 0·81 [95% CI 0·66–1·00], p=0·045) and subgroup analysis showed a larger effect for the node-positive subgroup. More precise prognostic or predictive classifications are urgently required^38^ so that the results of the de-escalation trials and those escalating treatment with dual HER2 therapy^37^ can be appropriately applied to optimise effectiveness while reducing toxicity and containing cost. As part of the translational research programme within the PERSEPHONE trial, over 80% of patients have donated formalin-fixed paraffin-embedded tumour tissue and germline blood, and our aim is to investigate whether it is possible to personalise trastuzumab duration taking into account germline and tumour genomics, as well as efficacy, toxicity, and standard prognostic factors.

In general, very substantial challenges remain to de-escalating effective treatments that have been used as standard for many years. An understandable reluctance on the part of both oncology teams and their patients to consider a change to practice, which has been established since 2005, is expected, despite the potential benefit for the individual patient of reduced toxicity, length of treatment, and a more rapid return to normal life. We are convinced that the optimal approach to evaluate shorter durations is to use registration trials, and we would strongly encourage such testing for new targeted adjuvant cancer therapies. Although we have shown non-inferiority for trastuzumab in the population we tested, discussion and intense debate about our results is ongoing, including whether or not these are applicable in 2019 compared with 2007 when the study was designed, because of the changes in standard treatments for patients with HER2-positive breast cancer that have occurred. Duration questions within registration trials could only occur with substantial high-level international collaboration between the pharmaceutical industry, international academic groups, and governments or medicines approval bodies (such as the European Medicines Agency and the US Food and Drug Administration), with input from the wider cancer specialist teams and patients with cancer, as has been discussed by Ponde and colleagues.^39^ If shorter treatments are found to be non-inferior by agreed statistical criteria at the outset, then these treatments will become the standard of care on licensing. The escalating cost of effective novel anti-cancer treatments is rapidly becoming unsustainable even for wealthy nations, and we believe clinical trials designed to test the non-inferiority of shorter treatments should become one of the priorities in cancer research.

In conclusion, in the PERSEPHONE trial, we have shown non-inferiority for 6-month adjuvant trastuzumab compared with 12-month treatment in patients with HER2-positive early breast cancer. The observed absolute difference in the primary endpoint of disease-free survival at 4 years was only 0·4%. This result signals the potential of reducing treatment duration to 6 months and, thereby, toxicity and cost, while obtaining similar efficacy for at least some women with HER2-positive breast cancer. This trial provides a positive result and will stimulate substantial debate because it is the only reduced-duration study to show non-inferiority for shorter adjuvant trastuzumab treatment.

## Data sharing

Data collected within the PERSEPHONE study will be made available to researchers whose full proposal for their use of the data has been approved by the PERSEPHONE Trial Management Group and whose research group includes a qualified statistician. The data required for the approved, specified purposes and the trial protocol will be provided, after completion of a data sharing agreement. Data sharing agreements will be set up by the sponsors of the trial, the funders, the trial coordination centre, and the Trial Steering and Management Groups. The data will be made available 2 years after publication. Please address requests for data to persephone@live.warwick.ac.uk.
