# Supplementary material for: 6 versus 12 months of adjuvant trastuzumab for HER2-positive early breast cancer (PERSEPHONE): 4-year disease-free survival results of a randomised phase 3 non-inferiority trial
Source: Lancet. 2019 Jun 29;393(10191):2599–612. doi: 10.1016/S0140-6736(19)30650-6 (PMC6615016; doi:10.1016/S0140-6736(19)30650-6)
Supplement: Supplementary appendix [file mmc1.pdf]

THE LANCET

Supplementary appendix

This appendix formed part of the original submission and has been peer reviewed. We post it as supplied by the authors.

Supplement to: Earl HM, Hiller L, Vallier A-L, et al. 6 versus 12 months of adjuvant trastuzumab for HER2-positive early breast cancer (PERSEPHONE): 4-year disease-free survival results of a randomised phase 3 non-inferiority trial. *Lancet* 2019; published online June 6. http://dx.doi.org/10.1016/S0140-6736(19)30650-6.

Web Extra Material

Contents

Supplementary Figures and Tables 1
Tables 1
Figures 7
Contributors 16
Committee Members 16
Contributing sites and consultants 17
Trial Investigators/contributing sites, PIs, and patient numbers 20

Supplementary Figures and Tables

Tables

Supplementary Table 1: Trastuzumab treatment received prior to randomisation

Number of Trastuzumab cycles received prior to randomisation	12-month group (N=2045)	6-month group (N=2043)	Total (N=4088)
0	898 (44%)	888 (43%)	1786 (44%)
1	293 (14%)	289 (14%)	582 (14%)
2	207 (10%)	205 (10%)	412 (10%)
3	171 (9%)	142 (7%)	313 (8%)
4	109 (5%)	119 (6%)	228 (5%)
5	110 (5%)	121 (6%)	231 (6%)
6	95 (5%)	95 (5%)	190 (5%)
7	64 (3%)	76 (4%)	140 (3%)
8	65 (3%)	60 (3%)	125 (3%)
9	33 (2%)	48 (2%)	81 (2%)

Supplementary Table 2: Treatment timing information across chemotherapy types

		Anthracycline based	Taxane based	Anthracycline + Taxane based	No Taxane and no Anthracycline
Chemotherapy timing	Adjuvant	1647 (48%)	389 (11%)	1427 (41%)	5 (<1%)
	Neoadjuvant	53 (9%)	14 (2%)	553 (89%)	0 (0)
Trastuzumab timing	Concurrent	59 (3%)	345 (18%)	1499 (79%)	0 (0)
	Sequential	1641 (75%)	58 (3%)	481 (22%)	5 (<1%)

Supplementary Table 3: Changing characteristics of patients randomised into PERSEPHONE

	Year of randomisation									
%	2007	2008	2009	2010	2011	2012	2013	2014	2015	% over 2007-2015
Concurrent CT and trastuzumab	0	24	18	32	36	46	57	69	70	47
Sequential CT and trastuzumab	100	76	82	68	64	54	43	31	30	53
ER Negative	0	38	37	35	33	30	28	27	26	31
ER Positive	100	62	63	65	67	70	72	73	74	69
Anthracycline based CT	50	63	58	48	49	44	35	28	25	42
Taxane based CT	0	7	6	5	5	10	11	17	18	10
Anthracycline + Taxane based CT	50	30	36	47	46	46	54	55	57	48
No taxane and no anthracycline CT	0	0	0	<1	<1	0	0	0	0	<1
Adjuvant CT	100	90	90	87	87	86	81	81	80	85
Neoadjuvant CT	0	10	10	13	13	14	19	19	20	15
Total N	4	86	356	573	753	759	589	558	410	4088

Supplementary Table 4: Patient Information, split by concurrent/sequential chemotherapy and trastuzumab, N (%)

	Concurrent (n=1903)	Sequential (n=2185)	Total (n=4088)	p
Randomised Treatment				0·98
12 months	951 (50)	1094 (50)	2045 (50)	
6 months	952 (50)	1091 (50)	2043 (50)	
ER status *				0·65
Negative	596 (31)	669 (31)	1265 (31)	
Positive	1307 (69)	1516 (69)	2823 (69)	
Chemotherapy type *				
Anthracycline based	59 (3)	1641 (75)	1700 (42)	<0·0001
Taxane based	345 (18)	58 (3)	403 (10)	
Anthracycline + Taxane based	1499 (79)	481 (22)	1980 (48)	
No taxane and no anthracycline	0 (0)	5 (<1)	5 (<1)	
Chemotherapy timing *				<0·0001
Adjuvant	1412 (74)	2056 (94)	3468 (85)	
Neo-adjuvant	491 (26)	129 (6)	620 (15)	
Age at randomisation				
Median (range)	54 (23-82)	57 (23-83)	56 (23-83)	<0·0001
<35 years old	65 (3)	30 (2)	95 (2)	<0·0001
35 – 49 years old	567 (30)	542 (27)	1109 (27)	
50 – 59 years old	593 (31)	671 (31)	1264 (31)	
60 +	678 (36)	942 (40)	1620 (40)	

Nodal Status at surgery [of the 3468 adjuvant patients]				<0·0001
Negative	644 (46)	1378 (67)	2022 (58)	
1–3 nodes positive	509 (36)	456 (22)	965 (28)	
4+ nodes positive	240 (17)	215 (10)	455 (13)	
Unknown	19 (1)	7 (1)	26 (1)	
Tumour size ^ [of the 3468 adjuvant patients]				<0·0001
<=2cm	581 (41)	1050 (51)	1631 (47)	
>2 and <=5cm	688 (49)	876 (43)	1564 (45)	
>5cm	89 (6)	81 (4)	170 (5)	
Unknown	54 (4)	49 (2)	103 (3)	
Tumour Grade ^				0·09
I (well diff.)	28 (2)	35 (2)	63 (2)	
II (mod. diff.)	560 (29)	710 (32)	1270 (31)	
III (poor diff.)	1233 (65)	1386 (63)	2619 (64)	
Unknown	82 (4)	54 (3)	136 (3)	
Ethnicity				<0·0001
White	1469 (77)	1837 (84)	3306 (81)	
Asian	70 (4)	39 (2)	109 (3)	
Black	55 (3)	42 (2)	97 (2)	
Other	24 (1)	14 (<1)	38 (1)	
Unknown	285 (15)	253 (12)	538 (13)	
Menopausal status before chemotherapy				0·006
Pre	607 (32)	540 (25)	1147 (28)	
Peri	134 (7)	126 (6)	260 (6)	
Post	971 (51)	1243 (57)	2214 (54)	
Not assessable/Not available	191 (10)	276 (13)	467 (12)	
Reported prior use of cardiac medication				0·93
Yes	47 (2)	52 (2)	99 (2)	
No	1856 (98)	2133 (98)	3989 (98)	
IHC[+]-score and FISH† positivity				0·003
3+	1401 (74)	1546 (71)	2947 (72)	
2+ and FISH† positive	437 (23)	600 (27)	1037 (25)	
Not available	65 (3)	39 (2)	104 (3)	
Median (IQR) Follow-Up (years)	4·5 (3·3-5·9)	5·8 (4·5-7·4)	5·4 (3·6-6·7)	
Number of deaths reported	122 (6%)	213 (10%)	335 (8%)	
Number of DFS events reported	205 (11%)	307 (14%)	512 (13%)	

* Stratification variable
^ of largest invasive tumour at diagnosis
+ IHC = Immunohistochemistry
† FISH = Fluorescence in situ hybridization
IQR = inter-quartile range
DFS events = disease-free survival events (a recorded relapse or death)

Supplementary Table 5: Reporting of trastuzumab treatment delays

	12-month group		6-month group	
**Reporting of treatment delays**	**In months 1-6**	**In months 7-12**	**In months 1-6**	**In months 7-12**
Cycles	1323/16687 (8%)	1059/15107 (7%)	1185/17119 (7%)	64/719 (9%)
Patients	860/1894 (45%)	706/1764 (40%)	795/1939 (41%)	46/93 (49%)

Supplementary Table 6: Cardiac Deaths Recorded in Clinical Record Forms

10 Cardiac Deaths recorded without metastatic disease
4 – Ischaemic heart disease – Unrelated / unlikely to be related
1 – LVEF decreased and CHF – Unrelated / unlikely to be related
1 – Acute heart failure / left ventricular hypertrophy and mitral valve disease – Unrelated / unlikely to be related
1 - Cardiac amyloid – Unrelated / unlikely to be related
1 - Cardiac sarcoid – Unrelated / unlikely to be related
1 - RV cardiomyopathy – Unrelated / unlikely to be related
1 – Death after routine CT scan – clinically anaphylaxis – no sign of this at post mortem – no cardiac pathology found.

3 deaths with metastatic disease were recorded as contributed to by cardiac problems
1 had controlled NYHA Type II CHF – Unrelated / unlikely to be related
1 had cardiomyopathy – Unrelated / unlikely to be related
1 had brain metastases and died during a seizure – Unrelated / unlikely to be related

TNO	Metastatic Y/N	Treatment Group	Trastuzumab doses	Time from diagnosis to death (months)	Cardiac causes	Comment
144	N	12m	18	44	Ischaemic heart disease	Unrelated/Unlikely. Death due to acute MI 44 months after diagnosis. No plausible association between trastuzumab and coronary artery disease
938	N	12m	8	21	Ischaemic heart disease	Unrelated/Unlikely. Death due to acute MI 21 months after diagnosis. No plausible association between trastuzumab and coronary artery disease
1754	N	12m	18	51	Cardiac amyloid	Unrelated - No plausible association between trastuzumab and amyloidosis
2003	N	12m	14	29	Cardiac sarcoid	Unrelated - No plausible association between trastuzumab and sarcoidosis
3021	N	12m	18	46	Right Ventricular arrhythmic cardiomyopathy	Unlikely - Arrhythmic cardiomyopathy (ARVC in old terminology) has a known genetic basis (mostly desmosomal genes) with physiological triggers (e.g. extreme endurance sports). It is plausible that cardiac dysfunction caused by trastuzumab could have been a trigger but unlikely in this case as heart function had recovered long before the ARVC developed.
3463	N	6m	9	53	Acute heart failure / Left ventricular hypertrophy / mitral valve disease	Unrelated/unlikely - there is no known association with left ventricular hypertrophy or mitral valve disease and it's difficult to think of a mechanism for any effect months after the discontinuation of trastuzumab.
35	N	6m	9	111	Ischaemic heart disease	Unrelated/Unlikely. Death due to acute MI 111(!) months after diagnosis. No plausible association between trastuzumab and coronary artery disease
1051	N	6m	4	78	CHF and type II respiratory failure	Unrelated/unlikely - Very unlikely to be related to trastuzumab - received only 4 doses, recovery of LV function >3 months after cessation, diagnosis of COPD, died 78 months after diagnosis
1086	N	6m	9	43	Not cardiac	N/A
2654	N	6m	9	41	Myocardial infarction – pulmonary embolism – atrial myxoma	Unrelated – no effect of trastuzumab during treatment, no association between trastuzumab and atrial myxoma or myocardial infarction
104	Y	12m	18	39	No cardiac problems – brain metastases – died during a seizure	N/A
625	Y	12m	3	35	Controlled NYHA II CHF	Death due to metastatic disease. LVEF recorded as 48% and trastuzumab stopped but no details of pre- LVEF or recovery of function so difficult to comment. An otherwise healthy woman unlikely to be symptomatic from an LVEF of 48% - was there something else going on? If she had symptomatic LV dysfunction this could have been caused by cancer treatment but an anthracycline effect is much more likely >1y after completion of trastuzumab

1279	Y	12m	4	75	Cardiomyopathy	Clear effect of trastuzumab causing reduced LVEF during treatment but death 6 years later not plausibly related to trastuzumab. No supportive information for cardiomyopathy - no histology. Possibly related to anthracycline.

Supplementary Table 7: Adverse Events and Cardiac Monitoring, over the two 6-month periods, for the 44% patients randomised prior to starting Trastuzumab

	12-month group			6-month group		
**Number of patients reporting at least one incidence of -**	**Overall**	**In months 1-6**	**In months 7-12**	**Overall**	**In months 1-6**	**In months 7-12**
Adverse event with severe[&] CTCAE grade	231/855 (27%)	185/855 (22%)	121/791 (15%)	195/852 (23%)	194/852 (23%)	2/44 (5%)
SAR to trastuzumab [$]	34/897 (4%)	22/897 (2%)	12/884^ (1%)	18/886 (2%)	17/886 (2%)	2/870^ (0·2%)
Clinical cardiac dysfunction [#]	103/872 (12%)	73/872 (8%)	76/857 (9%)	65/870 (7%)	51/870 (6%)	42/833 (5%)
Stopped trastuzumab permanently due to cardiac toxicity	75/855 (9%)	33/855 (4%)	42/791 (5%)	32/852 (4%)	31/852 (4%)	1/44 (2%)
Cardiac death †	4/897	0/897	0/884^	3/886	0/886	0/870^
Cardiac death related to trastuzumab †	0/897	0/897	0/884^	0/886	0/886	0/870^
Low LVEF *	114/895 (13%)	69/895 (8%)	80/857 (9%)	86/888 (10%)	73/888 (8%)	39/776 (5%)
Significant falls in LVEF						
Absolute decrease of >=10% from baseline to <50%	84/869 (10%)	48/865 (6%)	59/834 (7%)	64/857 (7%)	50/855 (6%)	28/755 (4%)
LVEF<50% after a baseline of >=59%	58/869 (7%)	30/865 (3%)	43/834 (5%)	47/857 (5%)	38/855 (4%)	19/755 (3%)

[&] CTCAE grade >=3, or 2 for palpitations
[$] Denominators exclude the 3 patients known not to have received Trastuzumab
^ Denominators reduced due to either deaths or withdrawal of consent for follow-up within the 1st 6 months.
[#] Clinical cardiac dysfunction = Symptoms of cardiac disease, and / or signs of congestive heart failure and / or new medication for cardiac disease
† 7 deaths were reported to have a ‘cardiac’ cause, either first cause or contributory. None occurred during the first 12 months after starting trastuzumab treatment. 6 patients died with no metastatic disease, and 1 had metastatic disease. In all cases trastuzumab was judged to be unrelated / unlikely to be related to cardiac problems
* Low LVEF = Number of patients with at least one LVEF<50%, or LVEF % unknown but classified on report as abnormal
Abbreviations:
SAR – Serious Adverse Reaction
LVEF = Left Ventricular Ejection Fraction

Figures

Supplementary Figure 1: Forest plots of Overall Survival for all patients (Panel A) and adjuvant only patients (Panel B)

a) OS – all patients

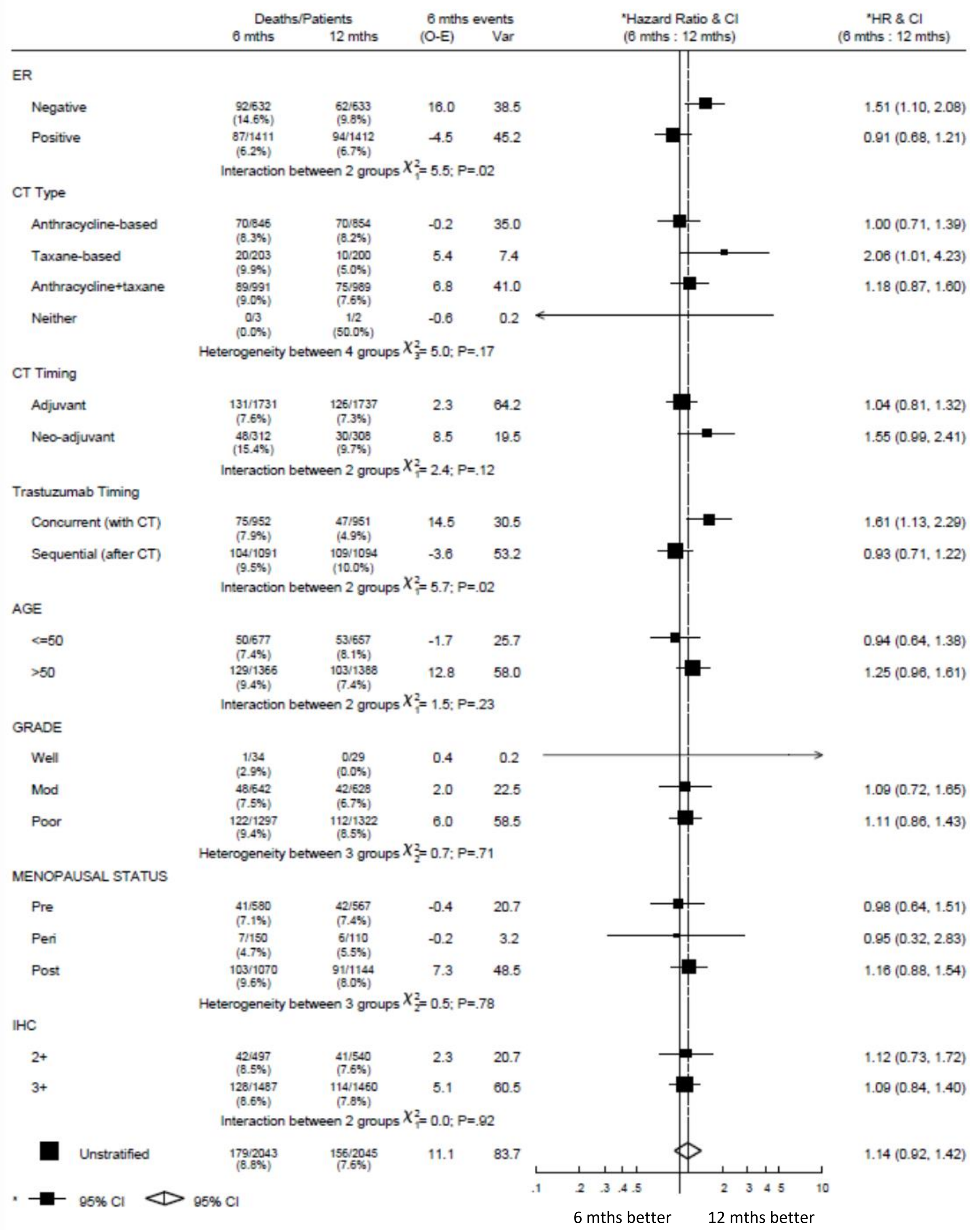

b) OS – adjuvant patients only

	Deaths/Patients 6 mths	Deaths/Patients 12 mths	6 mths events (O-E)	6 mths events Var	*HR & CI (6 mths : 12 mths)
**ER**					
Negative	62/504 (12.3%)	47/522 (9.0%)	8.9	27.2	1.38 (0.95, 2.02)
Positive	69/1227 (5.6%)	79/1215 (6.5%)	-6.0	37.0	0.85 (0.62, 1.17)
Interaction between 2 groups χ^2_1= 3.7; P=.05					
**CT Type**					
Anthracycline-based	59/819 (7.2%)	66/828 (8.0%)	-3.8	31.2	0.89 (0.62, 1.26)
Taxane-based	20/194 (10.3%)	9/195 (4.6%)	5.9	7.2	2.28 (1.10, 4.73)
Anthracycline+taxane	52/715 (7.3%)	50/712 (7.0%)	1.1	25.5	1.04 (0.71, 1.54)
Neither	0/3 (0.0%)	1/2 (50.0%)	-0.6	0.2	
Heterogeneity between 4 groups χ^2_3= 6.8; P=.08					
**Trastuzumab Timing**					
Concurrent (with CT)	44/708 (6.2%)	30/704 (4.3%)	7.0	18.5	1.46 (0.93, 2.31)
Sequential (after CT)	87/1023 (8.5%)	96/1033 (9.3%)	-4.8	45.7	0.90 (0.67, 1.20)
Interaction between 2 groups χ^2_1= 3.1; P=.08					
**NODE**					
NEG	53/1019 (5.2%)	41/1003 (4.1%)	5.6	23.5	1.27 (0.85, 1.90)
1-3	35/486 (7.2%)	33/479 (6.9%)	0.1	16.9	1.01 (0.62, 1.62)
4+	42/211 (19.9%)	52/244 (21.3%)	-2.7	23.4	0.89 (0.59, 1.34)
Heterogeneity between 3 groups χ^2_2= 1.5; P=.47					
**SIZE**					
<=2cm	36/807 (4.5%)	36/824 (4.4%)	0.0	18.0	1.00 (0.63, 1.59)
>2 - 5cm	72/786 (9.2%)	70/778 (9.0%)	1.1	35.5	1.03 (0.74, 1.43)
>5cm	19/83 (22.9%)	17/87 (19.5%)	0.3	8.7	1.03 (0.53, 2.01)
Heterogeneity between 3 groups χ^2_2= 0.0; P=.99					
**ER and Node**					
ER + NODE -	28/720 (3.9%)	27/711 (3.8%)	0.2	13.7	1.02 (0.60, 1.73)
ER - NODE -	25/299 (8.4%)	14/292 (4.8%)	5.4	9.7	1.74 (0.93, 3.26)
ER + NODE +	41/496 (8.3%)	52/497 (10.5%)	-6.5	23.2	0.75 (0.50, 1.13)
ER - NODE +	36/201 (17.9%)	33/226 (14.6%)	3.7	17.2	1.24 (0.77, 1.99)
Heterogeneity between 4 groups χ^2_3= 5.5; P=.14					
Unstratified	131/1731 (7.6%)	126/1737 (7.3%)	2.3	64.2	1.04 (0.81, 1.32)

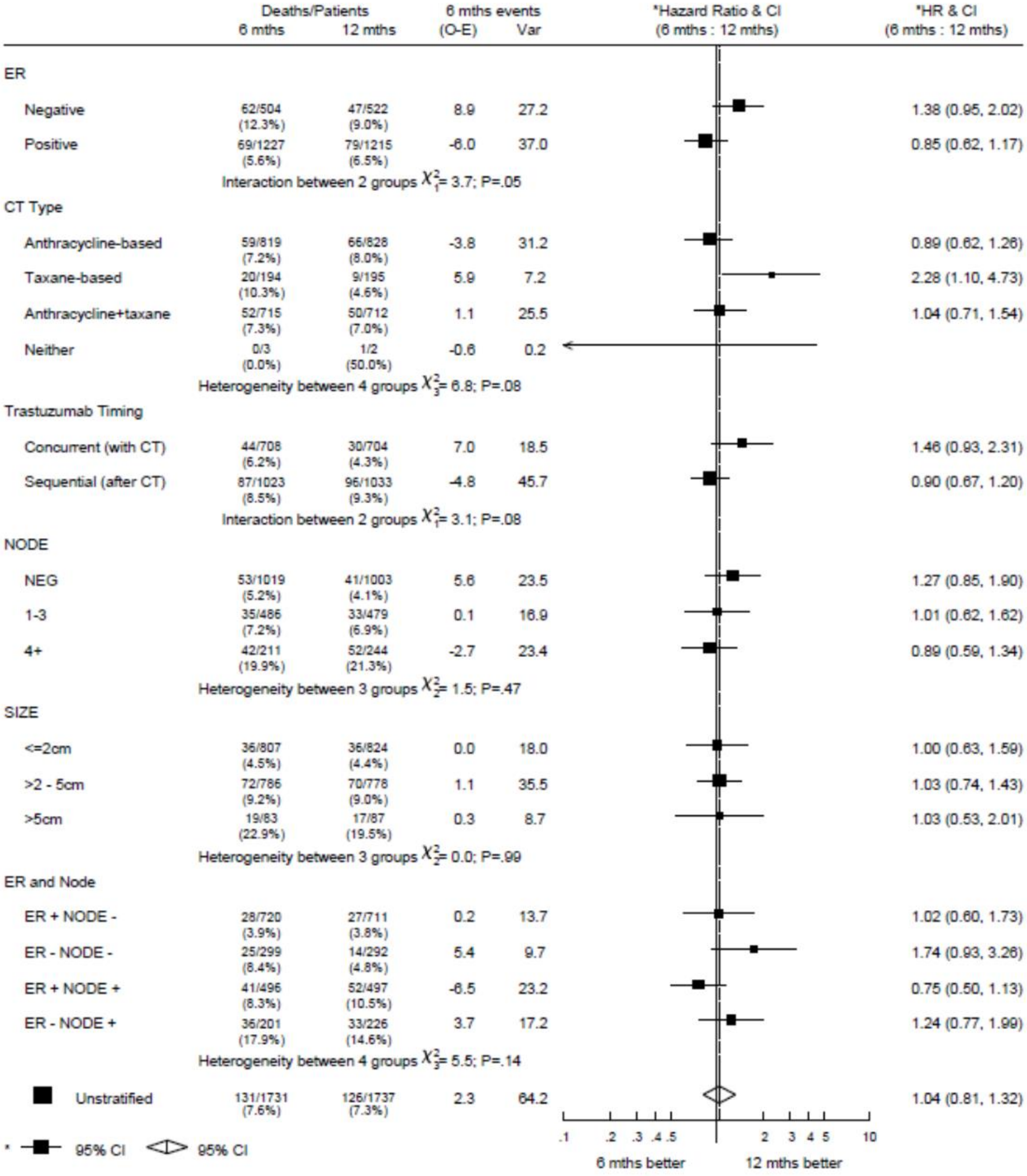

* ■ 95% CI ◇ 95% CI

Supplementary Figure 2: Forest plots of Landmark Disease-Free Survival (Panel A for all patients, Panel B for adjuvant patients only) and landmark Overall Survival (Panel C for all patients, Panel D for adjuvant patients only)

a) Landmark DFS – all patients

	Events/Patients 6 mths	Events/Patients 12 mths	6 mths events (O-E)	6 mths events Var	*HR & CI (6 mths : 12 mths)
**ER**					
Negative	116/620 (18.7%)	95/619 (15.3%)	12.0	52.7	1.26 (0.96, 1.65)
Positive	133/1380 (9.6%)	139/1390 (10.0%)	-4.2	68.0	0.94 (0.74, 1.19)
Interaction between 2 groups χ^2_1= 2.5; P=.11					
**CT Type**					
Anthracycline-based	86/828 (10.4%)	101/841 (12.0%)	-7.9	46.7	0.85 (0.63, 1.13)
Taxane-based	25/197 (12.7%)	10/196 (5.1%)	7.8	8.7	2.46 (1.27, 4.77)
Anthracycline+taxane	138/972 (14.2%)	122/970 (12.6%)	8.1	65.0	1.13 (0.89, 1.45)
Neither	0/3 (0.0%)	1/2 (50.0%)	-0.6	0.2	
Heterogeneity between 4 groups χ^2_3=10.4; P=.02					
**CT Timing**					
Adjuvant	182/1695 (10.7%)	189/1712 (11.0%)	-3.7	92.7	0.96 (0.78, 1.18)
Neo-adjuvant	67/305 (22.0%)	45/297 (15.2%)	11.3	28.0	1.50 (1.03, 2.17)
Interaction between 2 groups χ^2_1= 4.2; P=.04					
**Trastuzumab Timing**					
Concurrent (with CT)	115/930 (12.4%)	81/933 (8.7%)	18.3	49.0	1.45 (1.10, 1.92)
Sequential (after CT)	134/1070 (12.5%)	153/1076 (14.2%)	-11.1	71.7	0.86 (0.68, 1.08)
Interaction between 2 groups χ^2_1= 8.1; P=.004					
**Age**					
<=50	87/659 (13.2%)	84/640 (13.1%)	0.8	42.7	1.02 (0.75, 1.37)
>50	162/1341 (12.1%)	150/1369 (11.0%)	6.5	78.0	1.09 (0.87, 1.36)
Interaction between 2 groups χ^2_1= 0.1; P=.73					
**Grade**					
Well	2/34 (5.9%)	0/29 (0.0%)	0.9	0.5	
Mod	74/633 (11.7%)	68/621 (11.0%)	1.8	35.5	1.05 (0.76, 1.46)
Poor	162/1271 (12.7%)	163/1296 (12.6%)	1.2	81.2	1.01 (0.82, 1.26)
Heterogeneity between 3 groups χ^2_2= 1.7; P=.43					
**Menopausal Status**					
Pre	74/569 (13.0%)	68/554 (12.3%)	2.8	35.5	1.08 (0.78, 1.50)
Peri	11/148 (7.4%)	10/107 (9.3%)	-0.9	5.1	0.84 (0.36, 2.00)
Post	131/1051 (12.5%)	133/1129 (11.8%)	2.0	65.9	1.03 (0.81, 1.31)
Heterogeneity between 3 groups χ^2_2= 0.3; P=.87					
**IHC**					
2+	56/486 (11.5%)	66/529 (12.5%)	-2.9	30.5	0.91 (0.64, 1.30)
3+	178/1461 (12.2%)	164/1438 (11.4%)	5.2	85.5	1.06 (0.86, 1.31)
Interaction between 2 groups χ^2_1= 0.5; P=.46					
Unstratified	249/2000 (12.5%)	234/2009 (11.6%)	7.6	120.7	1.06 (0.89, 1.27)

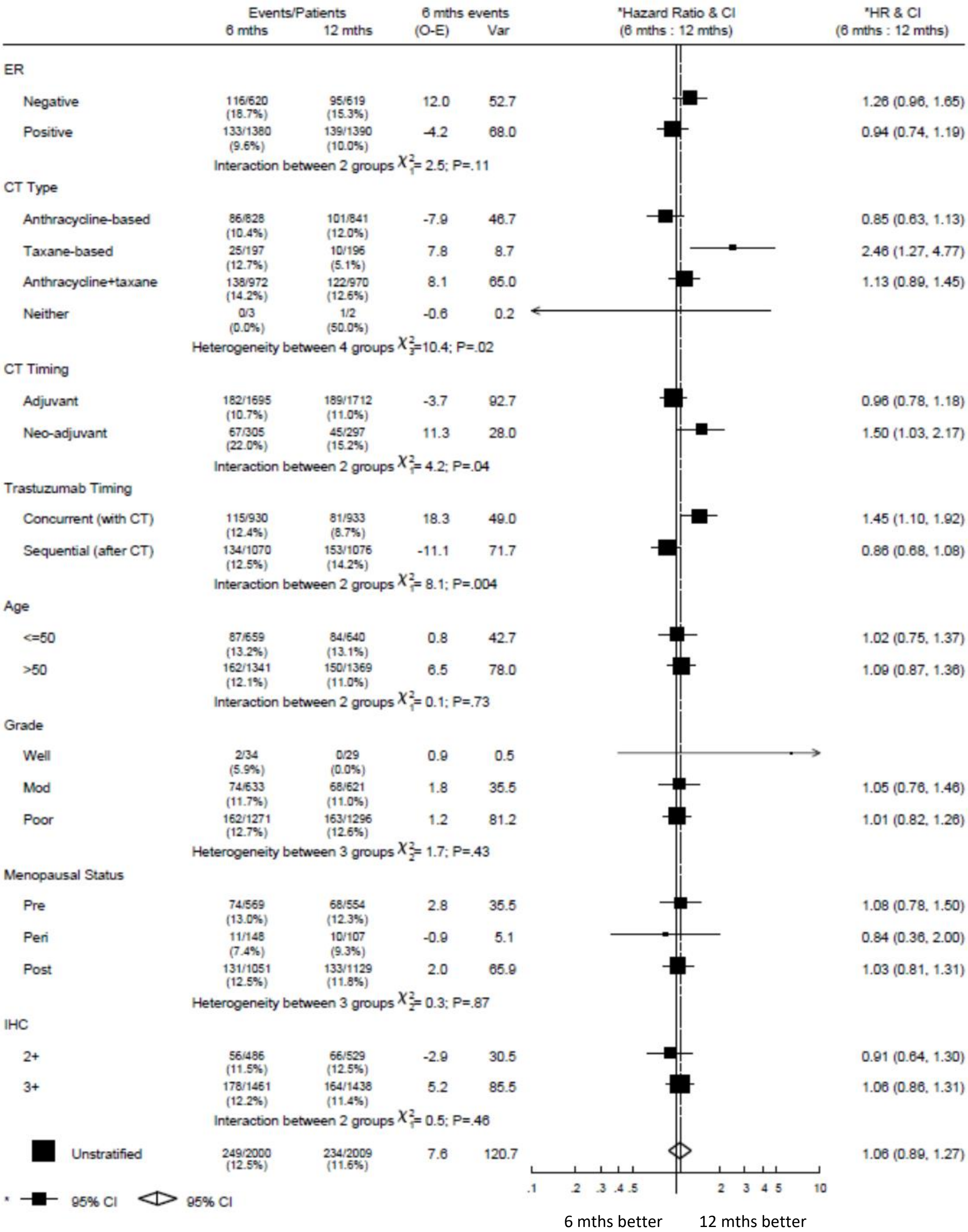

* 95% CI   95% CI

b) Landmark DFS – adjuvant patients only

	Events/Patients 6 mths	Events/Patients 12 mths	6 mths events (O-E)	6 mths events Var	*HR & CI (6 mths : 12 mths)
**ER**					
Negative	79/494 (16.0%)	75/511 (14.7%)	3.9	38.5	1.11 (0.81, 1.52)
Positive	103/1201 (8.6%)	114/1201 (9.5%)	-6.9	54.2	0.88 (0.67, 1.15)
Interaction between 2 groups χ^2_1= 1.2; P=.28					
**CT Type**					
Anthracycline-based	74/801 (9.2%)	96/816 (11.8%)	-11.5	42.5	0.76 (0.56, 1.03)
Taxane-based	24/188 (12.8%)	9/191 (4.7%)	7.9	8.2	2.63 (1.33, 5.20)
Anthracycline+taxane	84/703 (11.9%)	83/703 (11.8%)	0.5	41.7	1.01 (0.75, 1.37)
Neither	0/3 (0.0%)	1/2 (50.0%)	-0.6	0.2	
Heterogeneity between 4 groups χ^2_3=12.2; P=.007					
**Trastuzumab Timing**					
Concurrent (with CT)	72/693 (10.4%)	50/693 (7.2%)	11.5	30.5	1.46 (1.02, 2.08)
Sequential (after CT)	110/1002 (11.0%)	139/1019 (13.6%)	-15.3	62.2	0.78 (0.61, 1.00)
Interaction between 2 groups χ^2_1= 7.9; P=.005					
**Node**					
Neg	70/999 (7.0%)	64/990 (6.5%)	2.6	33.5	1.08 (0.77, 1.52)
1-3	62/478 (13.0%)	53/473 (11.2%)	3.4	28.7	1.12 (0.78, 1.62)
4+	49/208 (23.6%)	72/240 (30.0%)	-8.8	30.0	0.75 (0.52, 1.07)
Heterogeneity between 3 groups χ^2_2= 3.1; P=.21					
**Size**					
<=2cm	55/793 (6.9%)	61/814 (7.5%)	-2.7	29.0	0.91 (0.63, 1.31)
>2 - 5cm	100/772 (13.0%)	105/767 (13.7%)	-2.6	51.2	0.95 (0.72, 1.25)
>5cm	22/81 (27.2%)	20/86 (23.3%)	0.7	10.5	1.07 (0.58, 1.96)
Heterogeneity between 3 groups χ^2_2= 0.2; P=.90					
**ER and Node**					
ER + Node -	37/708 (5.2%)	38/703 (5.4%)	-0.9	18.7	0.95 (0.61, 1.50)
ER - Node -	33/291 (11.3%)	26/287 (9.1%)	3.8	14.7	1.30 (0.78, 2.16)
ER + Node +	66/487 (13.6%)	76/492 (15.4%)	-6.4	35.5	0.84 (0.60, 1.16)
ER - Node +	45/199 (22.6%)	49/221 (22.2%)	0.5	23.4	1.02 (0.68, 1.53)
Heterogeneity between 4 groups χ^2_3= 2.1; P=.56					
Unstratified	182/1695 (10.7%)	189/1712 (11.0%)	-3.7	92.7	0.96 (0.78, 1.18)

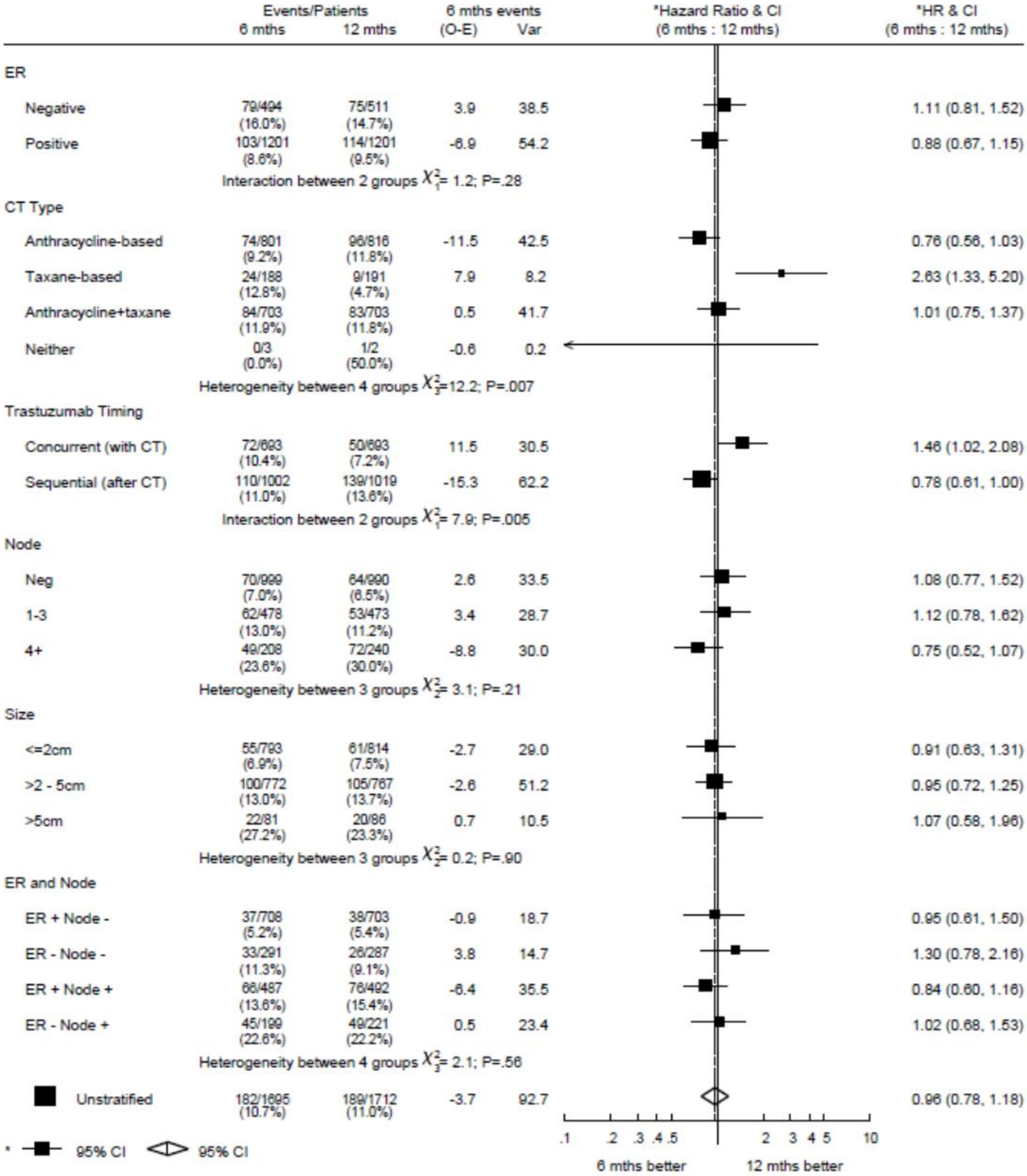

* 95% CI 95% CI

c) Landmark OS – all patients

	Death/Patients 6 mths	Death/Patients 12 mths	6 mths events (O-E)	6 mths events Var	*HR & CI (6 mths : 12 mths)
**ER**					
Negative	88/620 (14.2%)	59/619 (9.5%)	15.3	36.7	1.52 (1.10, 2.09)
Positive	78/1380 (5.7%)	87/1390 (6.3%)	-5.4	41.2	0.88 (0.65, 1.19)
Interaction between 2 groups χ^2_1= 5.8; P=.02					
**CT Type**					
Anthracycline-based	63/828 (7.6%)	65/841 (7.7%)	-1.2	32.0	0.96 (0.68, 1.36)
Taxane-based	17/197 (8.6%)	9/196 (4.6%)	4.3	6.5	1.94 (0.90, 4.19)
Anthracycline+taxane	86/972 (8.8%)	71/970 (7.3%)	7.5	39.2	1.21 (0.89, 1.65)
Neither	0/3 (0.0%)	1/2 (50.0%)	-0.6	0.2	
Heterogeneity between 4 groups χ^2_3= 4.5; P=.21					
**CT Timing**					
Adjuvant	121/1695 (7.1%)	120/1712 (7.0%)	0.4	60.2	1.01 (0.78, 1.30)
Neo-adjuvant	45/305 (14.8%)	26/297 (8.8%)	9.1	17.7	1.67 (1.05, 2.66)
Interaction between 2 groups χ^2_1= 3.5; P=.06					
**Trastuzumab Timing**					
Concurrent (with CT)	69/930 (7.4%)	46/933 (4.9%)	12.1	28.7	1.53 (1.06, 2.20)
Sequential (after CT)	97/1070 (9.1%)	100/1076 (9.3%)	-2.6	49.2	0.95 (0.72, 1.25)
Interaction between 2 groups χ^2_1= 4.1; P=.04					
**Age**					
<=50	45/659 (6.8%)	46/640 (7.2%)	-0.7	22.7	0.97 (0.64, 1.46)
>50	121/1341 (9.0%)	100/1369 (7.3%)	10.4	55.2	1.21 (0.93, 1.57)
Interaction between 2 groups χ^2_1= 0.8; P=.38					
**Grade**					
Well	1/34 (2.9%)	0/29 (0.0%)	0.4	0.2	
Mod	46/633 (7.3%)	41/621 (6.6%)	1.6	21.7	1.08 (0.71, 1.64)
Poor	111/1271 (8.7%)	103/1296 (7.9%)	5.0	53.5	1.10 (0.84, 1.44)
Heterogeneity between 3 groups χ^2_2= 0.7; P=.70					
**Menopausal Status**					
Pre	39/569 (6.9%)	37/554 (6.7%)	1.3	19.0	1.07 (0.68, 1.68)
Peri	7/148 (4.7%)	5/107 (4.7%)	0.3	2.9	1.09 (0.35, 3.43)
Post	95/1051 (9.0%)	89/1129 (7.9%)	4.4	46.0	1.10 (0.82, 1.47)
Heterogeneity between 3 groups χ^2_2= 0.0; P=.99					
**IHC**					
2+	37/486 (7.6%)	39/529 (7.4%)	0.5	19.0	1.03 (0.65, 1.61)
3+	120/1461 (8.2%)	106/1438 (7.4%)	5.4	56.5	1.10 (0.85, 1.43)
Interaction between 2 groups χ^2_1= 0.1; P=.80					
Unstratified	166/2000 (8.3%)	146/2009 (7.3%)	9.7	78.0	1.13 (0.91, 1.41)

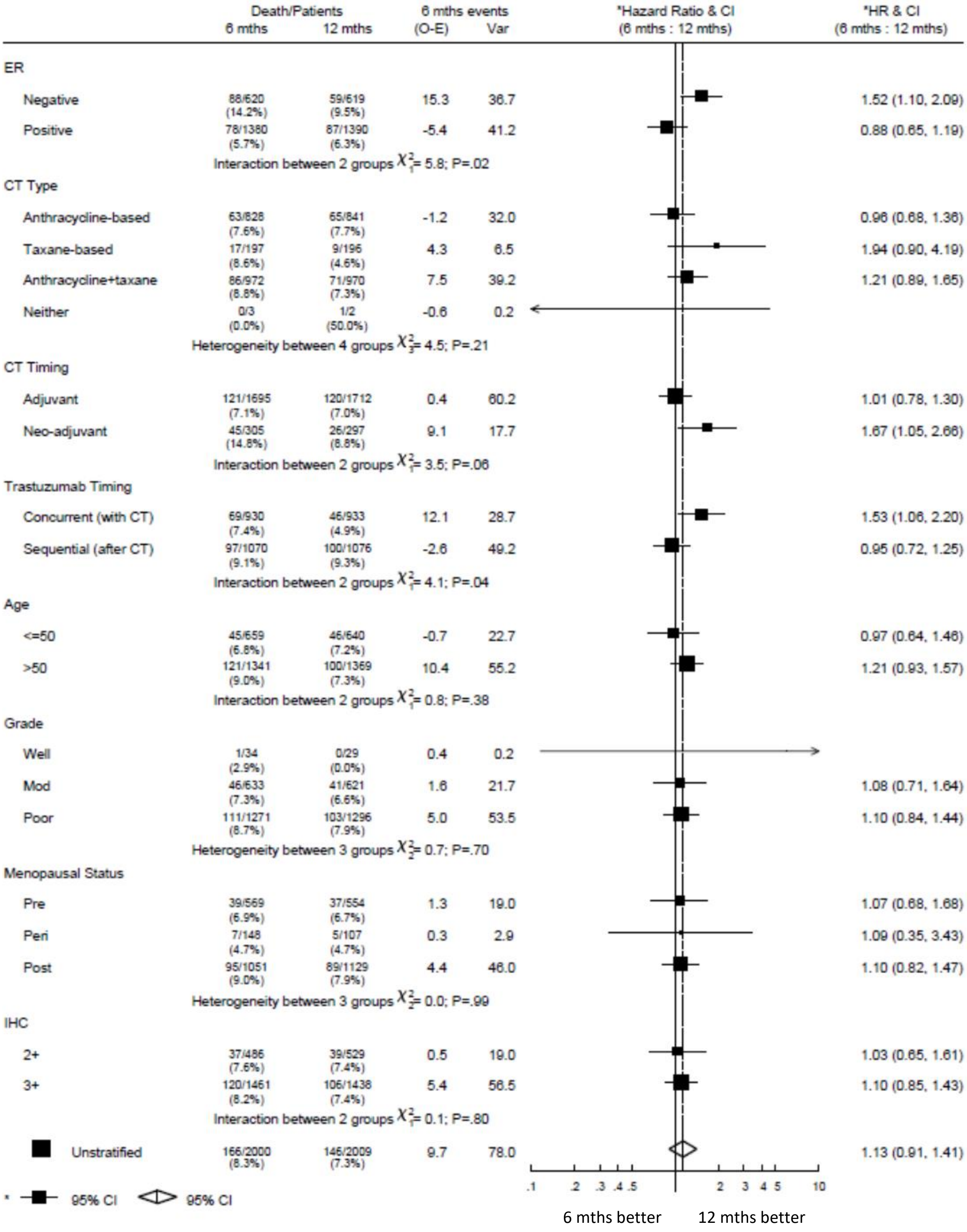

* ■ 95% CI ◇ 95% CI

d) Landmark OS – adjuvant patients only

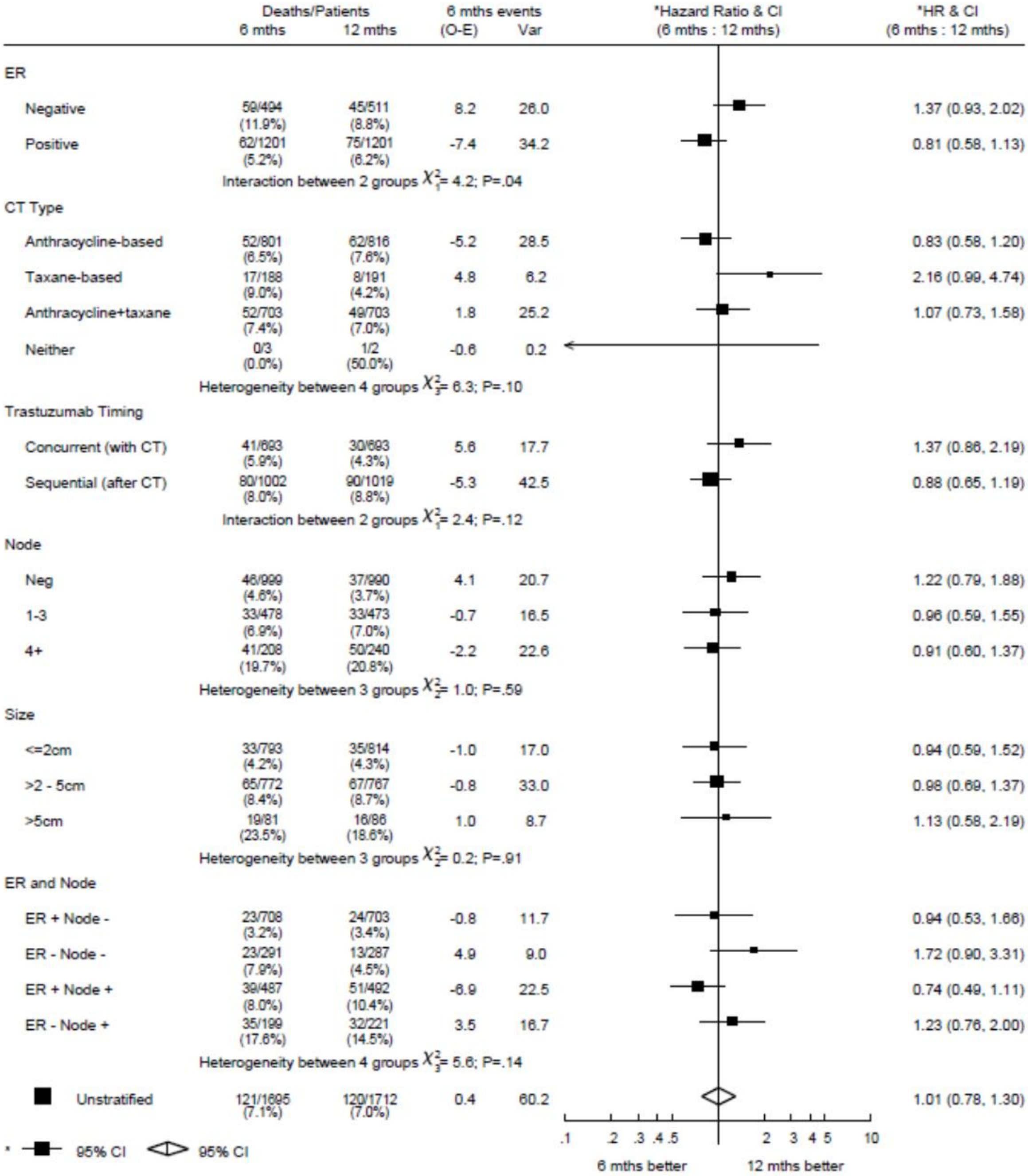

Supplementary Figure 3: Patients reporting an Adverse Event of severe grade (CTCAE>=3, or 2 for palpitations)

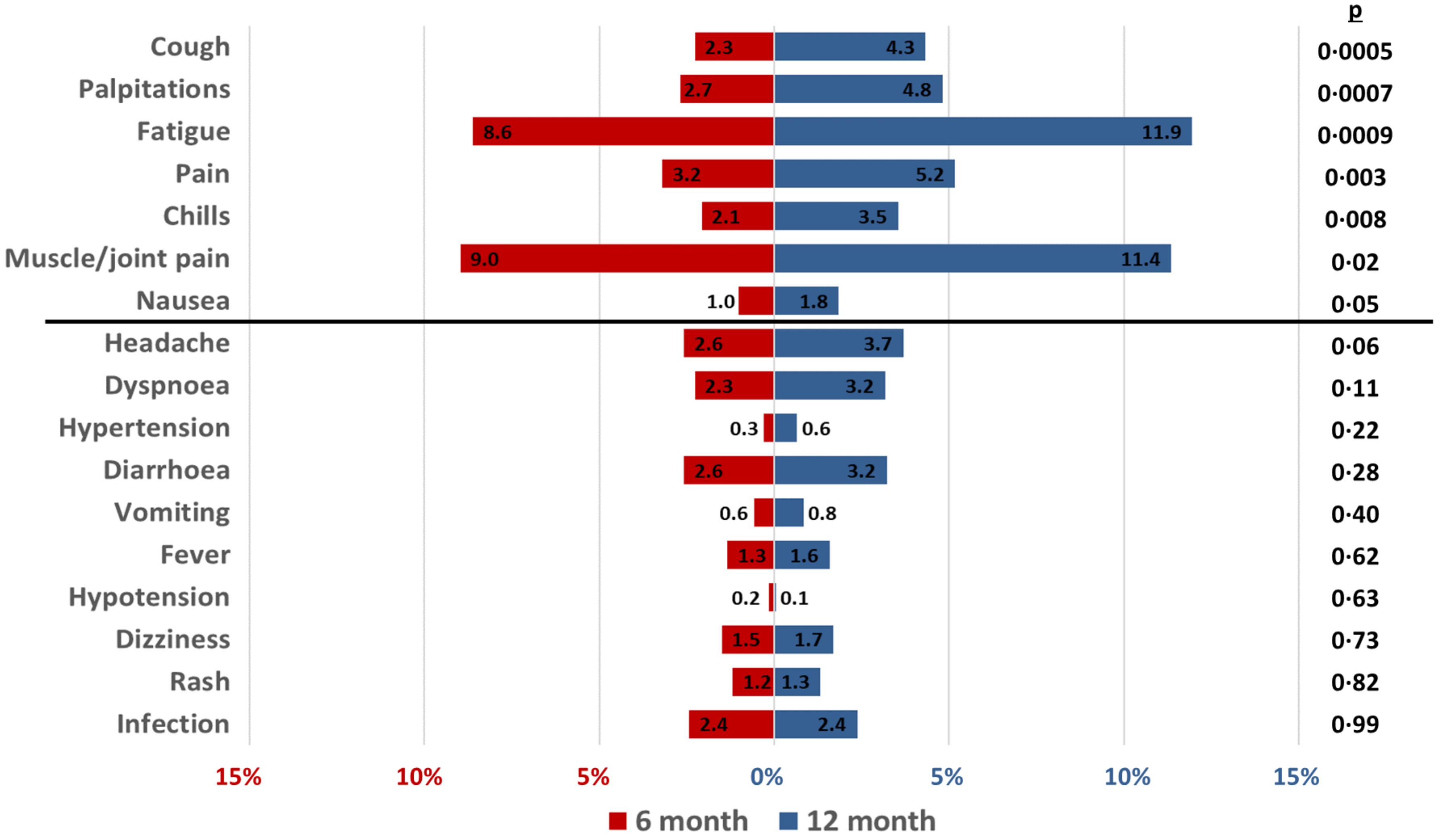

Supplementary Figure 4: Quality of Life over time; General Health (Panel A) and EQ-5D-3L Visual Analogue Scale (Panel B)

(A)

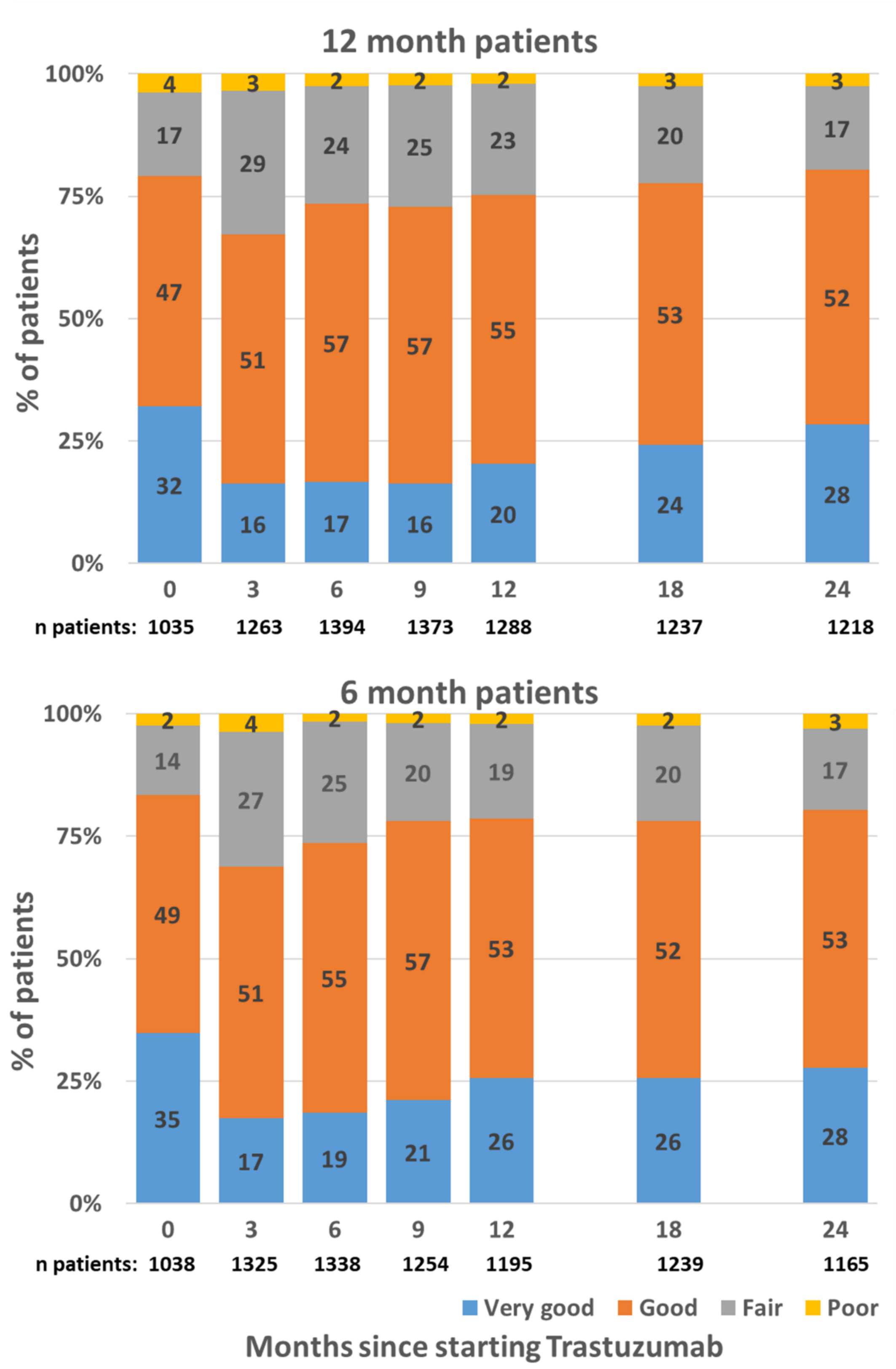

(B)

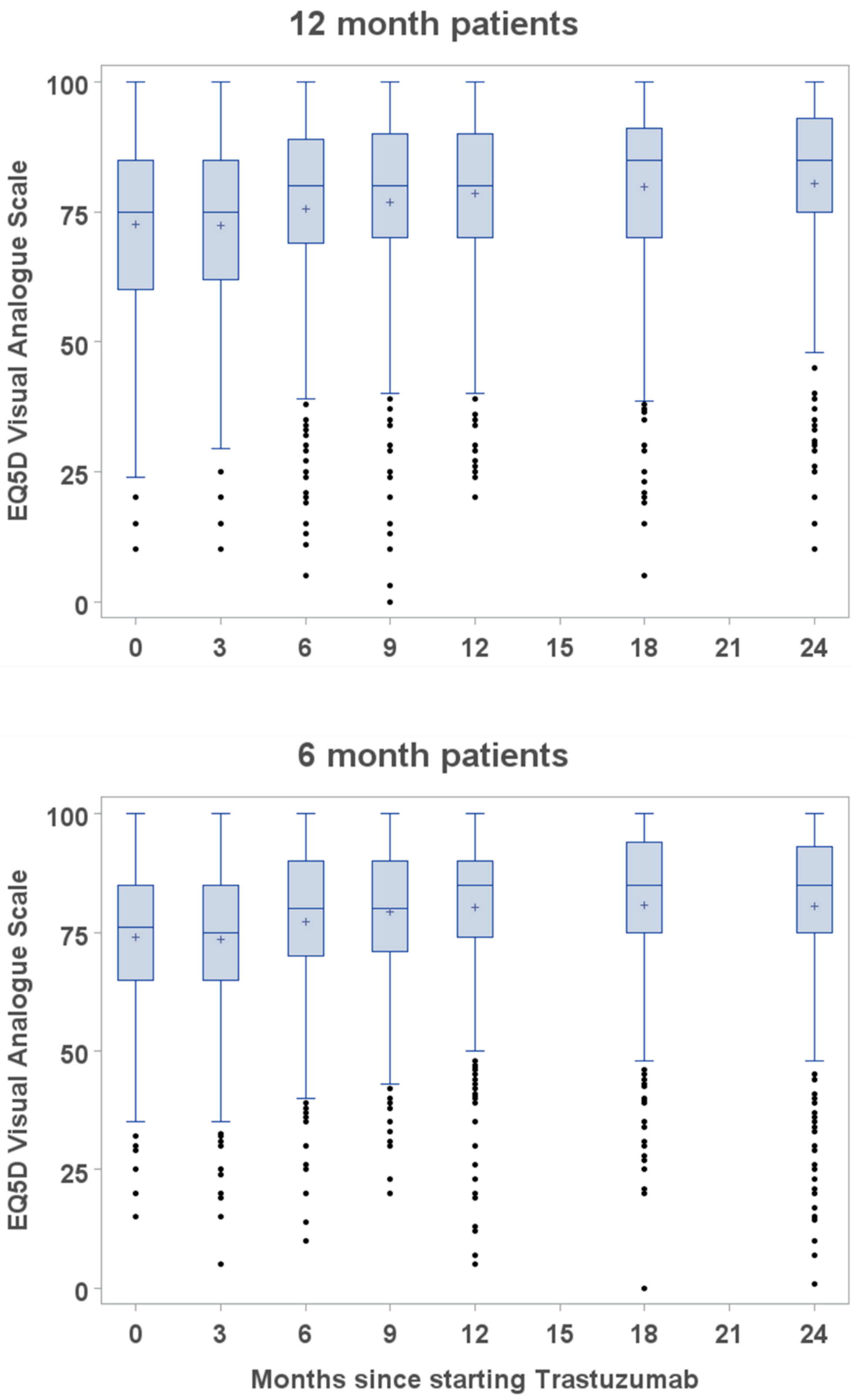

12 month patients
EQ5D Visual Analogue Scale
100
75
50
25
0
0
3
6
9
12
15
18
21
24
6 month patients
EQ5D Visual Analogue Scale
100
75
50
25
0
0
3
6
9
12
15
18
21
24
Months since starting Trastuzumab

Contributors

Committee Members

**Data Monitoring and Safety Committee**	
First Name	Last Name
Marianne	Nicolson
Richard	Bell
Roger	Ahern
**Independent Steering Committee**	
Adele	Francis (deceased)
Richard	Gray
Tim	Maughan
**Steering Committee**	
Lead Investigators	
Helena	Earl
David	Miles
David	Cameron
Andrew	Wardley
Clinical Co-ordinators	
Rajiv	Agrawal
Rozenn	Allerton
Peter	Bliss
Carlos	Caldas
Peter	Canney
Stephen	Chan
Michael	Crawford
Sue	Dean
Christopher	Gallagher
Rosalind	Glasspool
Adrian,	Harnett
Tamas	Hickish
Steve	Houston
Luke	Hughes-Davies
Alison	Jones
Robert	Leonard
Michael	Lind
Andreas	Makris
Karen	McAdam
Janine	Mansi
Susan	O’Reilly
Ann	Robinson
Timothy	Perren
Pippa	Riddle
Diana	Ritchie
Peter	Simmonds
Mark	Verrill
Charles	Wilson
Clinical surgical co-ordinators	
Gordon	Wishart
Anthony	Skene
Cardiology Advisor	
David	Dutka
Protocol authors	
Helena	Earl
Anne-Laure	Vallier
Janet	Dunn

Louise	Hiller
Chris	McCabe
Statisticians	
Janet	Dunn
Louise	Hiller
Trial Co-ordinators	
Shrushma	Loi
Anne-Laure	Vallier
Linda	Jones
Pharmacovigilance	
Helena	Earl
Anne-Laure	Vallier
Health Economics Advisor	
Christopher	McCabe
Phase III co-ordinating centre	
Warwick Medical School Clinical Trials Unit, University of Warwick	
Translational Sub-committee	
Carlos	Caldas
PHARE Group France	
Xavier	Pivot

Contributing sites and consultants

**Randomising Site**	**Randomising Consultant**
Aberdeen Royal Infirmary	Dr A Radha Todd, Dr Ravi Sharma, Dr Trevor McGoldrick
Addenbrooke's Hospital	Dr Charles Wilson, Dr Elizabeth Cox, Dr Jean Abraham, Dr Karen McAdam, Dr Luke Hughes-Davies
Airedale General Hospital	Dr Chris Bradley, Dr Shazza Rehman
Alexandra Hospital	Dr Clive Irwin
Ashford Hospital	Dr May Teoh
Barnet Hospital	Dr Kin Woo, Dr Peter Ostler, Dr Rob Stein
Barnsley District Hospital	Dr Bernadette Birtwhistle, Dr Caroline Lee, Dr Lucy Walkington
Basildon and Thurrock University Hospital	Dr Helen Swinburn, Dr Naveed Sarwar, Dr Wendy Ella
Basingstoke and North Hampshire Hospital	Dr Felicity Ross, Dr Sandra Tinkler
Bedford General Hospital	Dr Robert Thomas, Dr Sarah Smith
Birmingham Heartlands Hospital	Dr Indrajit Fernando
Bishop Auckland General Hospital	Dr Nicolas J Wadd
Blackpool Victoria Hospital	Dr Andrew Hindley, Dr Falalu Danwata, Dr Pavel Bezecny, Dr Shabbir Susnerwala
Borders General Hospital	Dr Carolyn Bedi
Bradford Royal Infirmary	Dr Chris Bradley, Dr Shazza Rehman
Broomfield Hospital	Dr Udaiveer Panwar, Dr Vivienne Loo, Prof Neville Davidson
Castle Hill Hospital	Dr Amandeep Dhadda, Dr Georgios Bozas, Dr Penny O'Neill, Dr Saiqa Spensley, Dr Sunil Upadhyay, Prof Michael Lind
Charing Cross Hospital	Dr Andrea Zivi, Dr Carlo Palmieri, Dr Charles Lowdell, Dr Conrad Lewanski, Dr Constantine Alifrangis, Dr Hanine Medani, Dr Laura Kenny, Dr Matthew Flook, Dr Sean O'Cathail, Dr Susan Cleator, Prof Charles Coombes, Prof Justin Stebbing, Prof Robert Leonard
Cheltenham General Hospital	Dr Jo Bowen, Dr Kim Benstead, Dr Peter Jenkins, Dr Radhika Counsell, Dr Roger Owen, Dr Sean Elyan
Chesterfield Royal Hospital	Dr Omar Din
Christie Hospital	Dr Abbas Chittalia, Dr Andrew Wardley, Dr Anne Armstrong, Dr Helen Mitchell, Dr Juliette Loncaster, Dr Sasha Howell
City Hospital	Dr Daniel Rea, Dr David Spooner
Clatterbridge Centre for Oncology	Dr Allison Hall, Dr Douglas Errington, Dr Farida Alam, Dr Khizar Hayat, Dr Susan O'Reilly, Dr Zafar Malik
Conquest Hospital,	Dr Craig Knighton, Dr Gillian Sadler
County Hospital	Dr Sam Guglani
Cumberland Infirmary	Dr Paul Dyson, Ms Helen Roe
Darent Valley Hospital	Dr Andrew Visioli, Dr Catherine Harper-Wynne, Dr Julia Hall

Darlington Memorial Hospital	Dr Alison Humphreys, Dr John Hardman, Dr Sophie Haney
Dewsbury and District Hospital	Dr Jay Naik
Diana, Princess of Wales Hospital	Dr Mohammad Butt, Dr Sunil Upadhyay
Doncaster Royal Infirmary	Dr Kathleen Dunn, Dr Sundareswaran Ramakrishnan
Dorset County Hospital	Dr Amitabha Chakrabarti, Dr Perric Crellin, Dr Susan Dean
Dumfries and Galloway Royal Infirmary	Dr Carolyn Bedi, Dr Marjory MacLennan, Dr Tamasin Evans
Ealing Hospital	Dr Conrad Lewanski
East Surrey Hospital	Dr Alexander Lee, Dr Eirini Thanopoulou, Dr Stephen Houston
Eastbourne District General Hospital	Dr Alistair Ring, Dr Charlotte Moss, Dr Sarah Westwell
Essex County Hospital	Dr Devy Basu, Dr Philip Murray, Dr Vivienne Loo
Freeman Hospital	Dr Mark Verrill
Friarage Hospital	Dr Hans Van der Voet, Dr Sarah Lawless
Furness General Hospital	Dr Geraldine Skailes, Dr Sarah Moon
George Eliot Hospital	Dr Lydia Fresco, Dr Medy Tsalic, Dr Susan Lupton
Glan Clwyd Hospital	Dr Catherine Bale, Dr Jill Bishop, Dr Win Soe
Good Hope Hospital	Dr Andrea Stevens, Dr Medy Tsalic
Great Western Hospital	Dr Anne Kendall, Dr David Cole, Dr Kinnari Patel, Dr Shiroma De Silva-Minor
Guy's Hospital	Dr Eleni Karapanagiotou, Dr Janine Mansi, Dr Mark Harries, Dr Paul Ellis, Dr Sarah Harris
Halton General Hospital	Prof Peter Clark
Hexham General Hospital	Dr Anthony N Branson
Hinchingbrooke Hospital	Dr Cheryl Palmer, Dr Clary Evans, Dr Simon Russell
Ipswich Hospital	Dr Karen E Sherwin, Dr Ramachandran Venkitaraman
James Paget Hospital	Dr Adrian Harnett, Dr May Thu Han
Kent and Canterbury Hospital	Dr Carys Thomas, Dr Julia Hall, Dr Natasha Mithal
Kidderminster Hospital	Dr Mark Churn
King Edward VII Hospital	Dr Narottam Thanvi, Dr Ruth Davis
King's College Hospital	Dr Anne Rigg, Dr Fiona Castell, Dr Vasiliki Michalarea
King's Mill Hospital	Dr Sarah Khan, Dr Victoria Brown
Leighton Hospital	Dr Laura Horsley
Lincoln County Hospital	Dr Abhro Chaudhuri, Dr Elisabeth Murray, Dr Thiagarajan Sreenivasan
Lister Hospital	Dr Nihal Shah
Luton and Dunstable Hospital	Dr Mei-Lin Ah-See
Macclesfield District General Hospital	Dr Lisa Barraclough, Dr Mark Lawrence
Maidstone Hospital	Dr Catherine Harper-Wynne, Dr Charlotte Abson, Dr Rema Jyothirmayi, Dr Russell Burcombe
Manor Hospital	Dr Indrajit Fernando, Dr Suhail Anwar
Medway Maritime Hospital	Dr Charlotte Abson, Dr Maher Hadaki
Milton Keynes Hospital	Dr Hany Eldeeb, Dr Maria Karina, Dr S. Azhar Rizvi
Mount Vernon Hospital	Dr Amy Guppy, Dr Andreas Makris, Dr Arshi Denton, Dr David Miles, Dr Peter Ostler, Prof Jane Maher
Musgrove Park Hospital	Dr Emma Cattell, Dr Hilary Barlow, Dr John Graham, Dr Mohini Varughese, Dr Saiqa Spensley
Nevill Hall Hospital	Dr Nayyer Iqbal, Dr Simon Waters, Dr Theresa Howe
New Cross Hospital	Dr Caroline Brammer, Dr Georgi Georgiev, Dr Laura Pettit, Dr Mark Churn, Dr Prakash Ramachandra, Dr Rakesh Mehra
Newham General Hospital	Dr Chris Gallagher, Dr Karen Tipples, Dr Virginia Wolstenholme
Norfolk & Norwich University Hospital	Dr Adrian Harnett, Dr Andrew Bulman, Dr Daniel Epurescu, Dr Dinos Geropantas, Dr Susanna Alexander
North Devon District Hospital	Dr Kate Scatchard
North Middlesex Hospital	Dr Fharat Raja, Dr Jacqueline Newby, Dr Stephen Karp
North Tyneside General Hospital	Dr Anthony N Branson
Northampton General Hospital	Dr Craig Knighton, Dr Craig Macmillan, Dr Hany Eldeeb
Northwick Park Hospital	Dr Andreas Makris, Dr Arshi Denton
Nottingham City Hospital	Dr Sarah Khan, Dr Stephen Chan
Peterborough City Hospital	Dr Catherine Jephcott, Dr Karen McAdam, Dr Sarah Ayers
Pilgrim Hospital	Dr Chiara Intrivici, Dr Elisabeth Murray
Pinderfields Hospital	Dr Jay Naik
Poole Hospital	Dr Amitabha Chakrabarti, Dr Darcy Goode, Dr Joanne Brady, Dr Susan Dean
Prince Charles Hospital	Dr Helen Passant

Princess of Wales Hospital	Dr James Powell, Dr Rosie Stevens
Princess Royal University Hospital	Dr Elinor Sawyer, Dr Mark Harries
Queen Alexandra Hospital	Dr Caroline Archer, Dr Helen Cooper, Dr Joanna Gale, Dr Timothy Gulliford
Queen Elizabeth Hospital (Birmingham)	Dr Andrea Stevens, Dr Daniel Rea, Dr Suhail Anwar
Queen Elizabeth Hospital (Gateshead)	Dr Daniela Lee, Dr Goudarz Mazdai, Dr Helen Lucraft, Dr Helen Turnbull, Dr Nicola Cresti, Dr Prithvi Jampana, Dr Rebecca Goranova, Dr Wendy Taylor
Queen Elizabeth Hospital (King's Lynn)	Dr Athar Ahmad, Dr Ellen Gokkel, Dr Margaret Daly, Dr Nicola Ainsworth, Dr Shahzeena Aslam,
Queen Elizabeth Hospital (London)	Dr Bruce Bryant, Dr Hartmut Kristeleit, Dr Mark Harries
Queen Elizabeth The Queen Mother Hospital	Dr Carys Thomas, Dr Jane Brown, Dr Julia Hall, Dr Natasha Mithal, Dr Rohit Malde
Queen's Hospital (Burton)	Dr Ad Chetiyawardana, Dr Mojca Persic
Queen's Hospital (Romford)	Dr Anwar Al-Saffar, Dr Caroline Bridgewater, Dr Eliot Sims, Dr Elizabeth Croydon, Dr Emma Staples, Dr Mark Prentice, Dr Mary Quigley
Raigmore Hospital	Dr Alison Nicholls, Dr Carol MacGregor, Dr David Whillis, Dr Kay Kelly, Dr Marion Paterson, Dr Neil McPhail, Dr Pinelopi Gkogkou, Dr Prantik Das
Rotherham General Hospital	Dr Alex Bradshaw, Dr Bernadette Birtwhistle, Dr Matthew Hatton
Royal Albert Edward Infirmary	Dr Elena Takeuchi, Dr Gregory Wilson
Royal Berkshire Hospital	Dr Jocelyn Adams, Dr Madhumita Bhattacharyya, Dr Richard Brown
Royal Bournemouth Hospital	Dr Tamas Hickish
Royal Derby Hospital	Dr Mojca Persic, Dr Pamela Woodings, Dr Prabir Chakraborti
Royal Free Hospital	Dr Alison Jones, Dr Jacqueline Newby
Royal Glamorgan Hospital	Dr Jacinta Abraham
Royal Gwent Hospital	Dr Carys Morgan, Dr Chris Gaffney, Dr Mohammed Harb, Dr Robert Jones, Dr Simon Waters
Royal Hampshire County Hospital	Dr Nicholas Murray, Dr Sanjay Raj, Dr Virginia Hall
Royal Lancaster Infirmary	Dr David Eaton, Dr Sarah Moon
Royal Liverpool University Hospital	Dr Nicola Thorp, Dr Susan O'Reilly
Royal Shrewsbury Hospital	Dr Huzeifa Gadir, Dr Laura Pettit, Dr Rajiv Agrawal, Dr Sheena Khanduri
Royal Surrey County Hospital	Dr Anthony Neal, Dr Robert Laing, Dr Stephen Houston
Royal Sussex County Hospital	Dr Alistair Ring, Dr David Bloomfield, Dr Gargi Patel, Dr Richard Simcock, Dr Sarah Westwell
Royal United Hospital	Dr Abigail Jenner, Dr Hugh Newman, Dr Mark Beresford, Dr Rebecca Bowen, Dr Susan Masson, Dr Susana Mancero
Russells Hall Hospital	Dr Georgi Georgiev, Dr Laura Pettit, Dr Muhammad Habibullah Khan, Dr Prakash Ramachandra, Dr Rozenn Allerton
Salisbury District Hospital	Dr Clare Crowley, Dr Ellen Copson, Dr Jennifer Bradbury, Dr Melanie Harvey
Sandwell General Hospital	Dr David Spooner
Scarborough General Hospital	Dr Amandeep Dhadda
Scunthorpe General Hospital	Dr Saiqa Spensley
Solihull Hospital	Dr Medy Tsalic
South Tyneside District Hospital	Dr Goudarz Mazdai, Dr Helen Turnbull, Dr Nicola Cresti, Dr Prithvi Jampana
Southampton General Hospital	Dr Clare Crowley, Dr Ellen Copson, Dr Jennifer Marshall, Dr Nicholas Murray, Dr Peter Simmonds, Dr Sanjay Raj
Southend Hospital	Dr Anne Robinson, Dr Colin Trask, Dr Hafiz Algurafi, Dr Helena Nam
Southport and Formby District General Hospital	Dr Helen Neville-Webbe, Dr Khizar Hayat, Dr Nasim Ali
St Bartholomew's Hospital	Dr Chris Cottrill, Dr Chris Gallagher, Dr Emma Spurrell, Dr John Conibear, Dr Karen Tipples, Dr Nita Patel, Dr Rebecca Roylance, Dr Virginia Wolstenholme
St. George's Hospital	Dr Ciara O'Hanlon Brown, Dr Laura Assersohn, Dr Muireann Kelleher
St. Mary's Hospital (IOW)	Dr Jennifer Marshall
St. Mary's Hospital (London)	Dr Susan Cleator
Stepping Hill Hospital	Dr Abbas Chittalia
Stoke Mandeville Hospital	, Dr Andrew Theobald, Dr Anthony Kong, Dr Christopher Alcock, Dr Clare Jacobs, Dr Eleanor James, Dr Thinn Pwint
Sunderland Royal Hospital	Dr A Radha Todd, Dr Kathryn Wright, Dr Sanjoy Chatterjee
The County Hospital (Prev. Staffordshire General)	Dr Amjad Al-Niaimi, Dr Apurna Jegannathen, Dr Caroline Brammer, Dr Laura Pettit
The James Cook University Hospital	Dr Alison Humphreys, Dr Nicola Storey

The Tunbridge Wells Hospital (Prev. Kent & Sussex)	Dr Rema Jyothirmayi
The Whittington Hospital	Dr Alison Jones, Dr Emma Spurrell, Dr Mulyati Mohamed, Prof Jayant Vaidya
Torbay Hospital	Dr Andrew Goodman, Dr Peter Bliss
University Hospital	Dr Clive Irwin, Prof Chris Poole, Prof Robert Grieve
University Hospital Aintree	Dr Julie O'Hagan, Dr Peter Robson
University Hospital Of Hartlepool	Dr Adrian Rathmell, Dr Eleanor Aynsley, Dr Nicola Storey
University Hospital Of North Durham	Dr Wendy Taylor
University Hospital Of North Tees	Dr Janine Graham, Dr Nicola Storey
Velindre Hospital	Dr Helen Passant, Dr Jacinta Abraham, Dr Nayyer Iqbal, Dr Rosie Stevens
Wansbeck General Hospital	Dr Anthony N Branson, Dr Helen Turnbull, Dr Nicola Cresti, Dr Rebecca Goranova
Warrington Hospital	Prof Peter Clark
Warwick Hospital	Dr Nawaz Walji, Prof Robert Grieve
West Cumberland Hospital	Dr Paul Dyson
West Middlesex University Hospital	Dr Pippa Riddle, Dr Rizvana Ahmad
West Suffolk Hospital,	Dr Anne Margaret Moody, Dr Cathryn Woodward
Western General Hospital	Dr Angela Bowman, Dr Larry Hayward, Dr Olga Olkonomidou, Dr Peter Hall, Prof David Cameron
Weston Park Hospital	Dr Kash Purohit, Dr Matthew Hatton, Dr Matthew Winter, Prof Robert Coleman
Wexham Park Hospital	Dr Jocelyn Adams, Dr Narottam Thanvi, Dr Richard Ashford, Dr Ruth Davis
Whiston Hospital	Dr Helen Innes, Dr Rajamram Sripadam
William Harvey Hospital	Dr Julia Hall, Dr Natasha Mithal
Worcester Royal Infirmary	Dr Jo Bowen, Dr Radhika Counsell
Worthing Hospital	Dr Ashok Nikapota, Dr Rebecca Herbertson, Dr Sankha Mitra
Wrexham Maelor Hospital	Dr Audrey Champion, Dr Niladri Ghosal, Dr Win Soe
Wycombe General Hospital	Dr Andrew Weaver, Dr Bernadette Lavery, Dr Ketan Shah, Dr Pattie Beresford, Dr Thinn Pwint
Yeovil District Hospital	Dr Geoffrey Sparrow, Dr Julie Walther, Dr Urmila Barthakur
Ysbyty Gwynedd	Dr Catherine Bale, Dr Jill Bishop, Dr Rachel Williams

Trial Investigators/contributing sites, PIs, and patient numbers

Randomising Site	Current PI	Total Patients Randomised
Addenbrooke's Hospital	Dr Helena Earl	95
Peterborough City Hospital	Dr Karen McAdam	83
Norfolk & Norwich University Hospital	Dr Daniel Epurescu	78
Royal Sussex County Hospital	Dr David Bloomfield	74
St Bartholomew's Hospital	Dr Karen Tipples	65
Southampton General Hospital	Dr Peter Simmonds	61
Royal Derby Hospital	Dr Pamela Woodings	60
Southend Hospital	Dr Hafiz Algurafi	60
Queen's Hospital (Romford)	Dr Eliot Sims	58
Wexham Park Hospital	Dr Narottam Thanvi	58
Charing Cross Hospital	Dr Susan Cleator	57
Maidstone Hospital	Dr Catherine Harper-Wynne	57
New Cross Hospital	Dr Rakesh Mehra	56
Luton and Dunstable Hospital	Dr Anup Vinayan	55
Royal Shrewsbury Hospital	Dr Laura Pettit	55
Royal Liverpool University Hospital	Dr Susan O'Reilly	54
Russells Hall Hospital	Dr Rozenn Allerton	51
Cumberland Infirmary	Ms Helen Roe	50
Eastbourne District General Hospital	Dr Charlotte Moss	50
Christie Hospital	Dr Anne Armstrong	49
Queen's Hospital (Burton)	Dr Mojca Persic	48
Royal Hampshire County Hospital	Dr Sanjay Raj	47

Great Western Hospital	Dr Shiroma De Silva-Minor	46
Royal Lancaster Infirmary	Dr David Eaton	45
Barnet Hospital	Dr Peter Ostler	44
James Paget Hospital	Miss Sue Down	43
Medway Maritime Hospital	Dr Maher Hadaki	43
Princess Royal University Hospital	Dr Mark Harries	42
Cheltenham General Hospital	Dr Kim Benstead	41
City Hospital	Dr Mariam Jafri	41
Conquest Hospital	Dr Charlotte Moss	41
Diana, Princess of Wales Hospital	Dr Mohammad Butt	41
Manor Hospital	Dr Sundus Yahya	41
Mount Vernon Hospital	Dr David Miles	41
Raigmore Hospital	Dr Carol MacGregor	41
King's College Hospital	Dr Anne Rigg	40
Macclesfield District General Hospital	Dr Lisa Barraclough	39
Queen Elizabeth Hospital (Birmingham)	Dr Daniel Rea	39
Torbay Hospital	Dr Andrew Goodman	39
University Hospital Of North Durham	Dr Wendy Taylor	39
Warwick Hospital	Dr Nawaz Walji	38
Guy's Hospital	Dr Janine Mansi	37
The County Hospital (Prev. Staffordshire General)	Dr Apurna Jegannathen	37
St. George's Hospital	Dr Muireann Kelleher	36
Yeovil District Hospital	Dr Urmila Barthakur	35
Clatterbridge Centre for Oncology	Dr Zafar Malik	34
Queen Elizabeth Hospital (King's Lynn)	Dr Margaret Daly	34
Queen Elizabeth Hospital (London)	Dr Hartmut Kristeleit	34
Southport and Formby District General Hospital	Dr Helen Neville-Webbe	34
Stoke Mandeville Hospital	Dr Andrew Eichholz	34
Royal Free Hospital	Dr Jacqueline Newby	33
William Harvey Hospital	Dr Natasha Mithal	32
Darent Valley Hospital	Dr Catherine Harper-Wynne	31
North Middlesex Hospital	Dr Fharat Raja	31
Royal United Hospital	Dr Rebecca Bowen	31
Sunderland Royal Hospital	Dr Kathryn Wright	31
Chesterfield Royal Hospital	Dr Omar Din	29
Musgrove Park Hospital	Dr Mohini Varughese	28
Queen Alexandra Hospital	Dr Caroline Archer	28
Salisbury District Hospital	Mrs Catherine Reed	28
The James Cook University Hospital	Dr Alison Humphreys	28
Essex County Hospital	Dr Mukesh Mukesh	27
Kent and Canterbury Hospital	Dr Jane Brown	27
University Hospital Of North Tees	Dr Nicola Storey	27
West Suffolk Hospital	Dr Anne Margaret Moody	27
Ysbyty Gwynedd	Dr Catherine Bale	27
Bedford General Hospital	Dr Sarah Smith	26
Royal Gwent Hospital	Dr Simon Waters	26
University Hospital	Prof Robert Grieve	26
Kidderminster Hospital	Dr Mark Churn	25
Queen Elizabeth Hospital (Gateshead)	Dr Daniela Lee	25
Wycombe General Hospital	Dr Andrew Eichholz	25
Bradford Royal Infirmary	Dr Chris Bradley	24
St. Mary's Hospital (IOW)	Dr Jennifer Marshall	24
Whiston Hospital	Dr Helen Innes	24
Worthing Hospital	Dr Adrian Moss	24
Northampton General Hospital	Dr Roshan Agarwal	23
Aberdeen Royal Infirmary	Dr Trevor McGoldrick	22
Borders General Hospital	Dr Carolyn Bedi	22
County Hospital	Dr Daniel Nelmes	22

George Eliot Hospital	Dr Susan Lupton	22
Royal Surrey County Hospital	Dr Anthony Neal	22
St. Mary's Hospital (London)	Dr Susan Cleator	22
Dorset County Hospital	Dr Perric Crellin	21
East Surrey Hospital	Dr Chee Goh	21
Lister Hospital	Dr Nihal Shah	21
The Whittington Hospital	Prof Jayant Vaidya	21
Castle Hill Hospital	Dr Amandeep Dhadda	20
Halton General Hospital	Dr Joanne Cliff	20
Scarborough General Hospital	Dr Amandeep Dhadda	20
South Tyneside District Hospital	Dr Daniela Lee	20
Warrington Hospital	Dr Joanne Cliff	20
Western General Hospital	Dr Larry Hayward	20
Newham General Hospital	Dr Karen Tipples	19
University Hospital Aintree	Dr Julie O'Hagan	19
Barnsley District Hospital	Dr Shobha Silva	17
Blackpool Victoria Hospital	Dr Pavel Bezecny	17
Dumfries and Galloway Royal Infirmary	Dr Larry Hayward	17
North Tyneside General Hospital	Dr Mahtab Najibah	17
University Hospital Of Hartlepool	Dr Nicola Storey	17
Wrexham Maelor Hospital	Dr Win Soe	17
West Middlesex University Hospital	Dr Rizvana Ahmad	16
Broomfield Hospital	Prof Saad Tahir	15
Glan Clwyd Hospital	Dr Jill Bishop	15
Lincoln County Hospital	Dr Chiara Intrivici	15
Royal Berkshire Hospital	Dr Jocelyn Adams	15
Basildon and Thurrock University Hospital	Dr Helen Swinburn	14
Ipswich Hospital	Dr Karen E Sherwin	14
North Devon District Hospital	Dr Kate Scatchard	14
Queen Elizabeth The Queen Mother Hospital	Dr Natasha Mithal	14
Airedale General Hospital	Dr Shazza Rehman	13
Darlington Memorial Hospital	Dr Wendy Taylor	13
The Tunbridge Wells Hospital (Prev. Kent & Sussex)	Dr Catherine Harper-Wynne	13
Pinderfields Hospital	Dr Jay Naik	12
Poole Hospital	Dr Amitabha Chakrabarti	12
Royal Glamorgan Hospital	Dr Jacinta Abraham	12
Velindre Hospital	Dr Jacinta Abraham	12
Basingstoke and North Hampshire Hospital	Dr Sanjay Raj	11
Hinchingbrooke Hospital	Dr Cheryl Palmer	11
Milton Keynes Hospital	Dr Maria Karina	11
Wansbeck General Hospital	Dr Mahtab Najibah	11
Weston Park Hospital	Dr Matthew Winter	11
Worcester Royal Infirmary	Dr Mark Churn	11
Alexandra Hospital	Dr Mark Churn	10
Doncaster Royal Infirmary	Dr Lynda Wyld	10
Furness General Hospital	Dr David Eaton	10
Good Hope Hospital	Dr Medy Tsalic	10
Nottingham City Hospital	Dr Stephen Chan	10
Princess of Wales Hospital	Dr Richard Webster	10
Birmingham Heartlands Hospital	Dr Indrajit Fernando	8
King's Mill Hospital	Dr Sarah Khan	8
Pilgrim Hospital	Dr Chiara Intrivici	8
Ealing Hospital	Dr Olivia Hatcher	7
Northwick Park Hospital	Dr Andreas Makris	7
Royal Albert Edward Infirmary	Dr Elena Takeuchi	7
Royal Bournemouth Hospital	Dr Tamas Hickish	7
Nevill Hall Hospital	Dr Theresa Howe	6
Rotherham General Hospital	Dr Matthew Hatton	6

Scunthorpe General Hospital	Dr Waqas Ali	6
Ashford Hospital	Dr May Teoh	5
Bishop Auckland General Hospital	Dr Wendy Taylor	5
Prince Charles Hospital	Dr Jacinta Abraham	5
Solihull Hospital	Dr Medy Tsalic	5
Hexham General Hospital	Dr Mahtab Najibah	4
Sandwell General Hospital	Dr Mariam Jafri	4
Freeman Hospital	Dr Mark Verrill	3
Friarage Hospital	Dr Alison Humphreys	3
King Edward VII Hospital	Dr Narottam Thanvi	3
Dewsbury and District Hospital	Dr Jay Naik	1
Leighton Hospital	Dr Laura Horsley	1
Stepping Hill Hospital	Dr Abbas Chittalia	1
West Cumberland Hospital	Ms Helen Roe	1
